# Endoplasmic reticulum stress inhibits AR expression via the PERK/eIF2α/ATF4 pathway in luminal androgen receptor triple-negative breast cancer and prostate cancer

**DOI:** 10.1038/s41523-021-00370-1

**Published:** 2022-01-10

**Authors:** Xiaoli Li, Duanfang Zhou, Yongqing Cai, Xiaoping Yu, Xiangru Zheng, Bo Chen, Wenjun Li, Hongfang Zeng, Moustapha Hassan, Ying Zhao, Weiying Zhou

**Affiliations:** 1 Department of Pharmacology, College of Pharmacy, Chongqing Medical University, Chongqing, 400016 P.R. China; 2 Chongqing Key laboratory of Drug Metabolism, Chongqing, 400016 P.R. China; 3 Key laboratory for Biochemistry and Molecular Pharmacology of Chongqing, Chongqing, 400016 P.R. China; 4 Department of Pharmacy, Army Medical Center of PLA, Chongqing, 400042 P.R. China; 5 Department of Gastrointestinal Surgery, The First Affiliated Hospital of Chongqing Medical University, Chongqing, 400016 P.R. China; 6 Department of Pharmacy, The Third Affiliated Hospital of Chongqing Medical University (Gener Hospital), Chongqing, 401120 P.R. China; 7 Experimental Cancer Medicine, Division of Bio-molecular and Cellular Medicine (BCM), Department of Laboratory Medicine, Karolinska Institutet, Huddinge, 141 86 Stockholm Sweden

**Keywords:** Breast cancer, Targeted therapies, Transcriptional regulatory elements, Oncogenes, Breast cancer

## Abstract

Androgen receptor (AR) is an important prognostic marker and therapeutic target in luminal androgen receptor triple-negative breast cancer (LAR TNBC) and prostate cancer (PCa). Endoplasmic reticulum (ER) stress may activate the unfolded protein response (UPR) to regulate associated protein expression and is closely related to tumor growth and drug resistance. The effect of ER stress on AR expression and signaling remains unclear. Here, we focused on the regulation and underlying mechanism of AR expression induced by ER stress in LAR TNBC and PCa. Western blotting and quantitative RT-PCR results showed that AR expression was markedly decreased under ER stress induced by thapsigargin and brefeldin A, and this effect was dependent on PERK/eIF2α/ATF4 signaling activation. Chromatin immunoprecipitation-PCR and luciferase reporter gene analysis results showed that ATF4 bound to the AR promoter regions to inhibit its activity. Moreover, ATF4 overexpression inhibited tumor proliferation and AR expression both in vitro and in vivo. Collectively, these results demonstrated that ER stress could decrease AR mRNA and protein levels via PERK/eIF2α/ATF4 signaling in LAR TNBC and PCa. Targeting the UPR may be a treatment strategy for AR-dependent TNBC and PCa.

## Introduction

The androgen receptor (AR) is a nuclear receptor that functions as a transcription factor activated by the steroid hormone androgen. Upon binding of its androgen ligand, the complex is translocated to the nucleus, where it binds to DNA and stimulates the transcription of androgen-responsive genes^1–3^.

Breast cancer and prostate cancer (PCa) are typical hormone-dependent malignant tumors with high heterogeneity. AR is the primary factor driving all stages of PCa. Therefore, targeted suppression of AR expression is the primary treatment for PCa via surgical or medical castration with anti-androgens or luteinizing hormone-releasing hormone analogs (agonists or antagonists)^4–7^. Interestingly, some studies have shown that AR is also highly expressed in up to 70–90% of all breast cancer types, including as much as 30% of triple-negative breast cancers (TNBC) that are deficient in the expression of estrogen receptor α (ERα), progesterone receptor (PR) and HER2. Furthermore, studies have shown that AR-directed therapy induces tumor response^3,8–10^. A luminal androgen receptor (LAR) subtype of TNBC (LAR TNBC) is dependent on AR signaling and AR has become increasingly important as a prognostic marker and potential therapeutic target in precision medicine for this TNBC subtype^10,11^. Therefore, AR-targeted drugs have the potential to be used in AR^+^ PCa and LAR TNBC-specific precision treatments.

The endoplasmic reticulum (ER) is an essential cellular organelle for the production, processing, and transport of membrane-bound or secretory proteins and lipids. The accumulation of misfolded proteins in the ER disrupts ER homeostasis resulting in ER stress^12^. Cells exposed to ER stress trigger the unfolded protein response (UPR)^12,13^. In mammals, the UPR is initiated through the activation of three ER-resident sensors: inositol-requiring enzyme 1α (IRE1α), activating transcription factor 6 (ATF6), and protein kinase R (PKR)-like ER kinase (PERK)^14,15^. These signaling pathways clear misfolded proteins and restore ER homeostasis by weakening protein translation and transcription to relieve ER load, upregulating the expression of genes that are involved in increasing ER protein folding capacity (e.g., BiP), and activating the ER-associated degradation pathway if the protein cannot be refolded correctly^16,17^. Thus, ER stress response dysfunction is associated with various diseases including neurodegeneration, chronic obstructive pulmonary disease, fatty liver, diabetes, and cancers^17^. Moreover, the UPR has been reported to be involved in the resistance of breast cancer cells to radiotherapy and chemotherapy. Conversely, the UPR can promote breast cancer cell death if ER stress is excessive or maintained for a prolonged period^12^.

In this study, we investigated the regulation and underlying mechanism of AR expression induced by ER stress in LAR TNBC and PCa. We hope to clarify whether targeting UPR could be a strategy for the treatment of AR-dependent TNBC and PCa.

## Results

### ER stress decreased AR protein expression in LAR TNBC and PCa cell lines

**Fig. 1:**
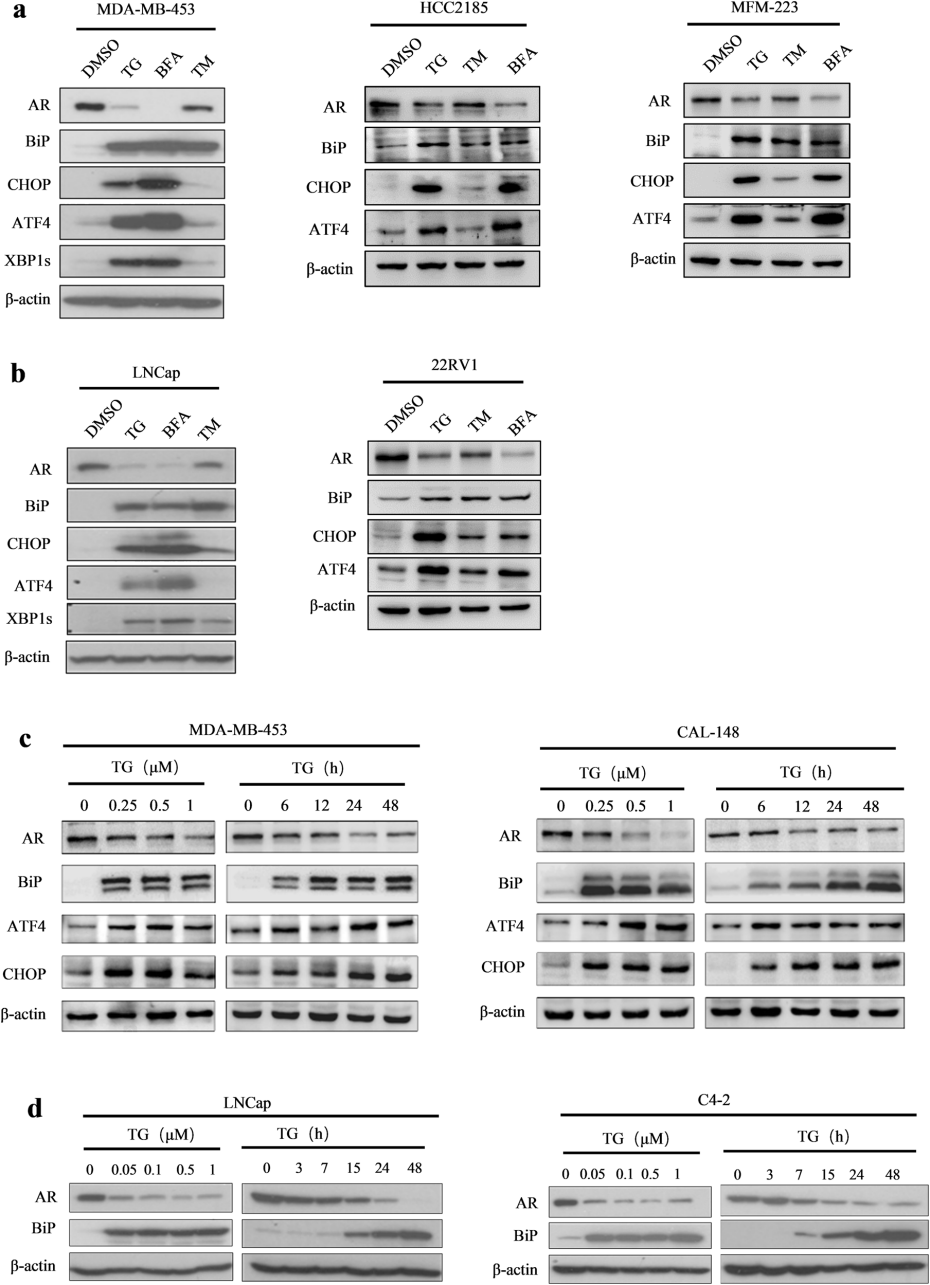
ER-stress inducers decreased AR protein expression in LAR TNBC and PCa cells. **a, b** MDA-MB-453, HCC2185, and MFM-223 cells (**a**), LNCap, and 22RV1 cells (**b**) were treated with 1 μM TG, 5 μg/mL BFA or 4 μg/mL TM for 24 h and collected for western blotting assay. **c**, **d** MDA-MB-453 and CAL-148 (**c**), LNCap and C4-2 (**d**) cells were treated with TG for 24 h or with 1 μM TG for the indicated time, and then cells were collected for western blotting assay. Thapsigargin, brefeldin A and tunicamycin are abbreviated as TG, BFA, and TM, respectively.

We first examined whether ER stress influences AR protein expression. LAR TNBC cell lines MDA-MB-453, HCC2185, and MFM-223, and PCa cell lines LNCap and 22RV1 were treated with ER stress inducers thapsigargin (TG), brefeldin A (BFA), and tunicamycin (TM) for 24 h, respectively. The levels of ER stress markers, immunoglobulin heavy chain-binding protein (BiP), and CCAAT/enhancer-binding protein homologous protein (CHOP) were markedly increased after treatment (Fig. 1a, b). The expression of ATF4 and XBP1s, markers of the PERK and IRE1 arms of the ER stress response, respectively, was also significantly increased after treatment with TG and BFA (Fig. 1a, b). By contrast, AR protein levels were significantly downregulated (Fig. 1a, b). Furthermore, TG decreased AR levels and increased the levels of BiP, CHOP, and ATF4 in a dose- and time-dependent manner in MDA-MB-453, CAL-148, LNCap, and C4-2 cell lines (Fig. 1c, d).

### ER stress downregulated AR expression at the transcriptional level in LAR TNBC and PCa cell lines

**Fig. 2:**
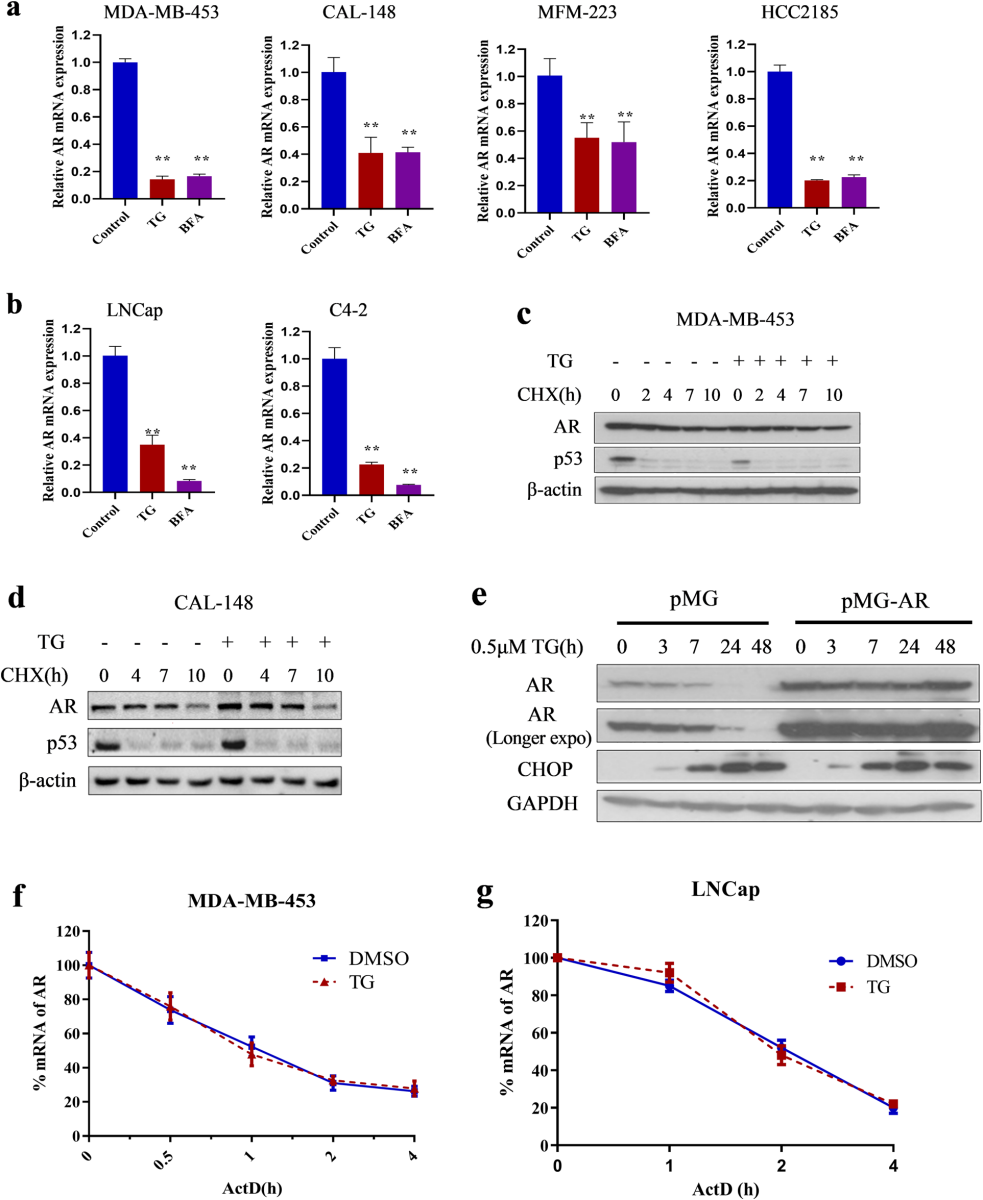
ER-stress down-regulated AR mRNA expression through transcriptional level in LAR TNBC and PCa cells. **a**, **b** Breast cancer cell lines MDA-MB-453, CAL-148, MFM-223 and HCC2185 (**a**), and prostate cancer cell lines LNCap and C4-2 (**b**) were treated with 1 μM TG or 5 μg/mL BFA for 24 h. Then cells were collected for quantitative RT-PCR assay. The relative expression of AR was normalized to β-actin and expressed as mean ± SD (*n* = 3). Student’s *t*-test, **p *<* 0.01 *vs*. control. **c**, **d** MDA-MB-453 (**c**) and CAL-148 (**d**) cells were pretreated with 100 μg/mL CHX for 1 h and then treated with or without 1 μM TG during the indicated times. After TG treatment, cells were collected for western blotting assays. **e** LNCap cells were transfected with empty vector (pMG) or overexpression of AR vector (pMG-AR) and then treated with or without TG during the indicated times. Cells were collected for western blotting assays. **f**, **g** MDA-MB-453 (**f**) and LNCap (**g**) cells were pretreated with 1 μg/mL ActD for 0.5 h and then treated with or without 1 μM TG during the indicated time. Then, cells were collected for quantitative RT-PCR assays (*n* = 3). Thapsigargin, cycloheximide, actinomycin D is abbreviated as TG, CHX, and ActD, respectively.

We examined the effects of ER stress on AR mRNA expression in LAR TNBC and PCa cell lines. TG and BFA increased the mRNA expression of BiP and CHOP (Supplementary Fig. 1) and decreased the mRNA expression of AR in MDA-MB-453, CAL-148, MFM-223, HCC2185, LNCap, and C4-2 cell lines (Fig. 2a, b). Subsequently, we investigated the mechanism by which ER stress decreased AR mRNA expression and, specifically, whether ER stress promoted AR protein and mRNA degradation. First, we examined the effects of ER stress on AR protein degradation using the protein synthesis inhibitor cycloheximide (CHX). AR protein levels decreased after CHX treatment in the presence or absence of TG, and no significant change in AR degradation rate was detected in the presence or absence of TG in MDA-MB-453 (Fig. 2c) and CAL-148 cells (Fig. 2d). Moreover, endogenous AR protein expression, rather than exogenous overexpressed AR protein gradually decreased after TG treatment in LNCap cells (Fig. 2e). These results indicated that TG did not affect AR protein stability or promote AR protein degradation. Subsequently, we examined the effects of ER stress on AR mRNA stability using the general transcription inhibitor actinomycin D (ActD). The results showed that TG did not affect the AR mRNA degradation rate in MDA-MB-453 and LNCap cells (Fig. 2f, g), suggesting that ER stress suppressed AR expression by reducing its mRNA synthesis rather than by increasing its degradation. Overall, these results indicated that ER stress downregulated AR expression at the transcriptional level.

### The decrease in AR levels induced by ER stress was dependent on the PERK/ATF4 pathway in LAR TNBC and PCa cell lines

**Fig. 3:**
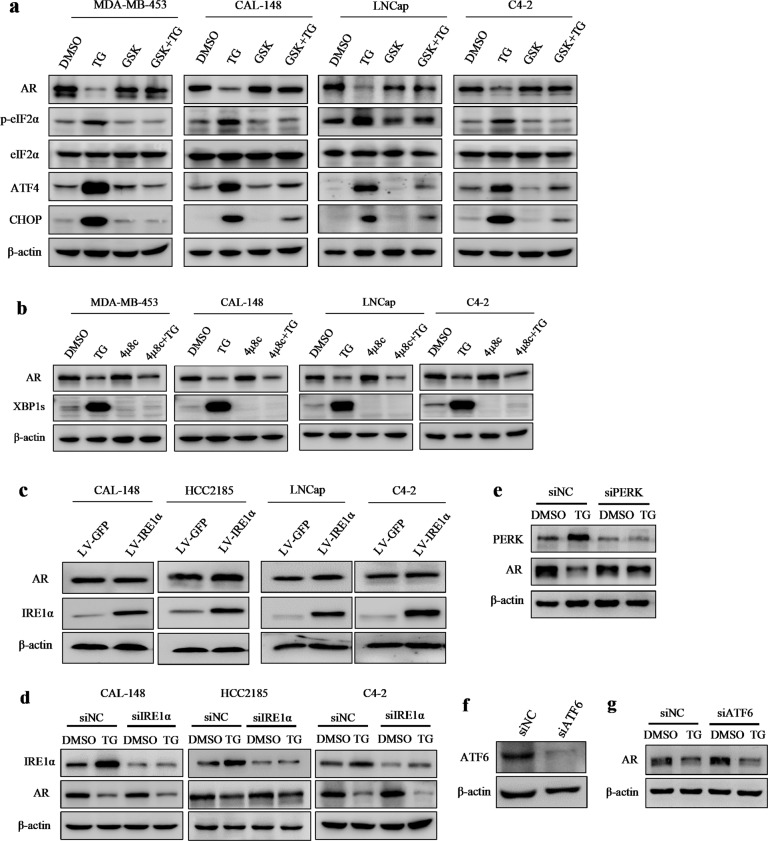
ER-stress decreased AR expression through PERK signaling in LAR TNBC and PCa cells. **a** MDA-MB-453, CAL-148, LNCap, and C4-2 cells were pretreated with 1 μM GSK2656157 (GSK) for 1 h and then treated with or without 1 μM TG for 24 h. Then cells were collected for western blotting assays. **b** MDA-MB-453, CAL-148, LNCap, and C4-2 cells were pretreated with 25 μM 4μ8C for 1 h and then treated with or without 1 μM TG for 24 h. Then cells were collected for western blotting assays. **c** CAL-148, HCC2185, LNCap, and C4-2 cells were transduced with negative control (LV-GFP) or IRE1α overexpression lentivirus (LV-IRE1α), then cells were collected for western blotting assays. **d** CAL-148, HCC2185, and C4-2 cells were transfected with control siRNA (siNC) or IRE1α siRNA (siIRE1α) for 12 h and then treated with or without TG for 24 h, the cells were collected for western blotting assays. **e–g** MDA-MB-453 cells were transfected with control siRNA (siNC), ATF6 siRNA(siATF6), or PERK siRNA(siPERK) for 12 h and then treated with or without TG for 24 h, the cells were collected for western blotting assays.

**Fig. 4:**
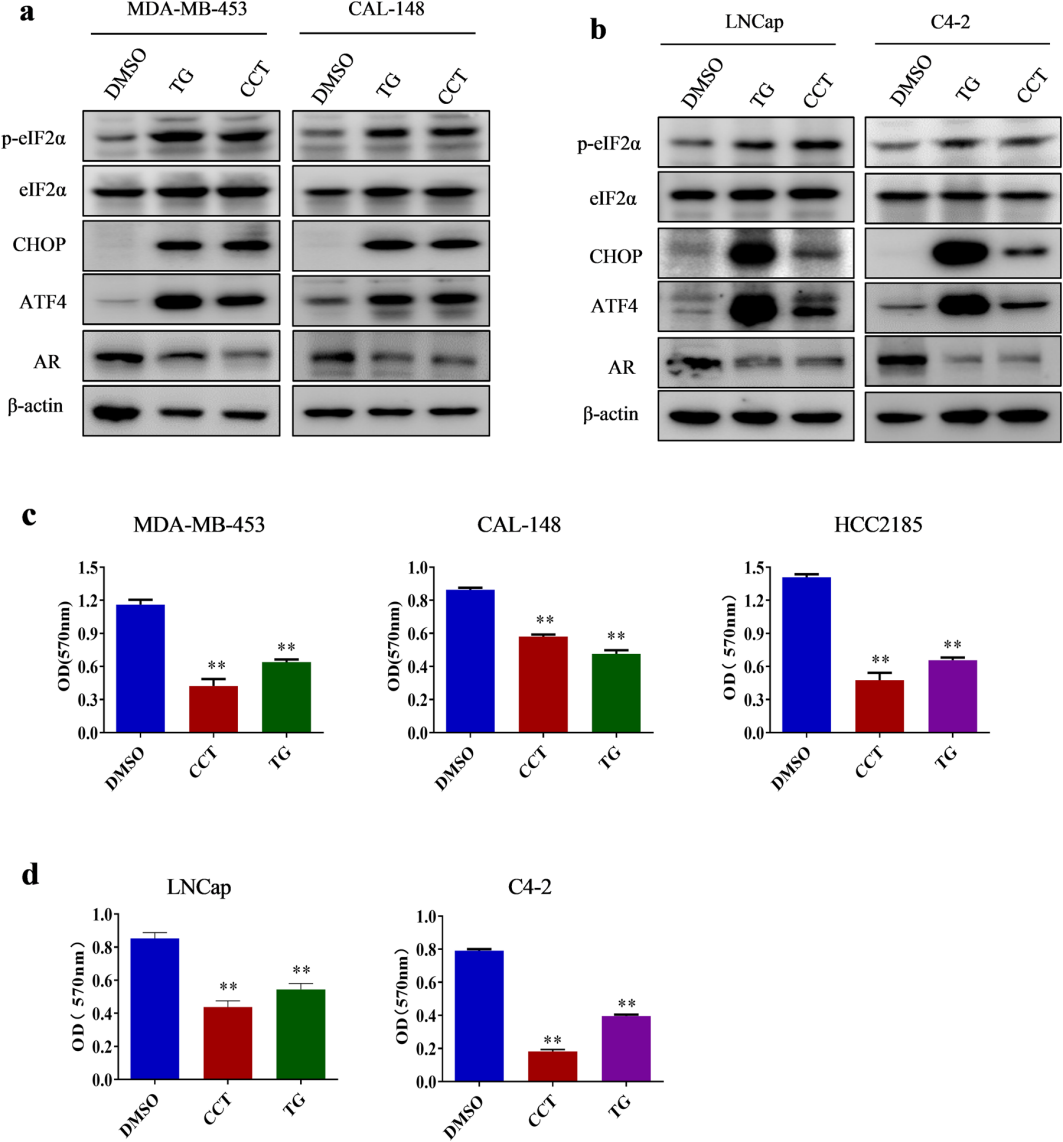
The selective eIF2α/PERK activator CCT020312 decreased AR expression and cell viability in LAR TNBC and PCa cells. **a, b** Breast cancer cell lines MDA-MB-453 and CAL-148 (**a**), and prostate cancer cell lines LNCap and C4-2 (**b**) were treated with 10 μM or 12 μM CCT020312 or 1 μM TG for 24 h. Then cells were collected for western blotting assays. **c, d** Breast cancer cell lines MDA-MB-453, CAL-148, HCC2185 (**c**), and prostate cancer cell lines LNCap and C4-2 (**d**) were treated with 10 μM or 12 μM CCT020312 or 1 μM TG for 24 h. Cells were collected for CCK-8 assays (*n* = 6). One-way ANOVA analysis, **p *<* 0.01 *vs*. DMSO.

The three main sensors—IRE1α, PERK, and ATF6—are activated during ER stress. To investigate which signaling pathway is responsible for AR downregulation by ER stress, we first utilized the commercially available inhibitors of IRE1 and PERK and examined their effects on ER stress-induced AR downregulation. In MDA-MB-453, CAL-148, LNCap, and C4-2 cells, TG increased the levels of PERK downstream of p-eIF2α, ATF4, and CHOP, whereas the PERK inhibitor GSK2656157 blocked the TG-induced increase in the expression of p-eIF2α, ATF4, and CHOP (Fig. 3a), establishing that GSK2656157 blocked TG-induced PERK activation. XBP1 is one of the most important IRE1 targets in the IRE1 pathway and is spliced into XBP1s once IRE1 is activated. TG dramatically increased the levels of XBP1s, whereas the IRE1 inhibitor 4μ8C blocked TG-induced increase in XBP1s levels (Fig. 3b), demonstrating that 4μ8C blocked IRE1 activity. Strikingly, pretreatment with GSK2656157, but not 4μ8C, effectively blocked TG-induced AR decrease (Fig. 3a, b), suggesting that the PERK pathway was involved in ER stress-induced AR downregulation. Furthermore, overexpression of IRE1α had no significant influence on AR expression (Fig. 3c), and knocking down PERK, but not IRE1α or ATF6, reversed the TG-induced AR decrease (Fig. 3d–g). These results further support the notion that the PERK pathway, but not the IRE1α or ATF6 pathways, participated in ER stress-induced AR decrease. To further verify that activation of the PERK pathway decreased AR expression, the selective eIF2α/PERK activator CCT020312 was used to treat breast cancer and PCa cells. As expected, CCT020312 activated PERK branches, as shown by increased levels of p-eIF2α, ATF4, and CHOP (Fig. 4a, b). By contrast, CCT020312 decreased AR levels in MDA-MB-453, CAL-148, LNCap, and C4-2 cells (Fig. 4a, b). Moreover, selective activation of PERK by CCT020312 also decreased cell viability in all the tested breast cancer and PCa cell lines (Fig. 4c, d).

**Fig. 5:**
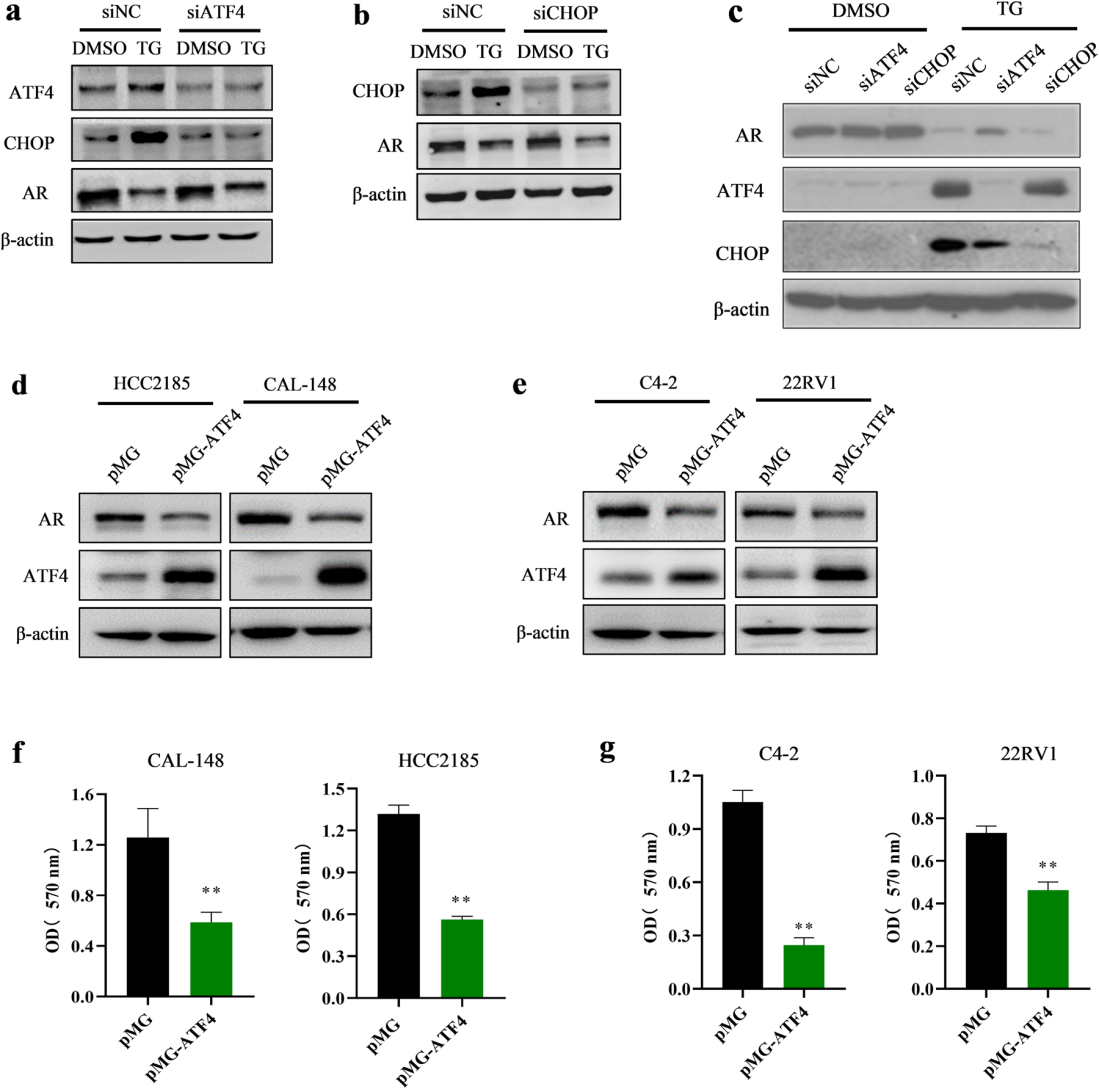
ER-stress decreased AR expression through ATF4 pathway in LAR TNBC and PCa cells. **a–c** MDA-MB-453 (**a**, **b**) and LNCap (**c**) cells were transfected with control siRNA (siNC), ATF4 siRNA (siATF4), or CHOP siRNA (siCHOP) for 12 h and then treated with 1 μM TG for 24 h. The cells were collected for western blotting assay. **d–g** HCC2185, CAL-148, C4-2, and 22RV1 cells were transfected with empty control plasmid (pMG) or ATF4 overexpression plasmid (pMG-ATF4). The transfected cells were collected for western blotting assays (**d**, **e**) or CCK-8 assays (*n* = 5) (**f**, **g**). Student’s *t*-test, **p *<* 0.01 *vs*. pMG.

Because ATF4 and CHOP are key transcription factors downstream of the PERK signaling pathway, we investigated whether they are involved in the regulation of AR expression. MDA-MB-453 cells were transfected with different siRNAs against ATF4 and CHOP to knock down their expression and stimulated with TG for 24 h. The results showed that transfection of MDA-MB-453 cells with ATF4 siRNA blocked TG-mediated increase in ATF4 expression and decrease in AR expression (Fig. 5a). However, transfection of MDA-MB-453 cells with CHOP siRNA blocked the TG-mediated increase in CHOP expression but did not block the TG-induced decrease in AR expression (Fig. 5b). Similar results were observed in the LNCap cells (Fig. 5c). These findings indicated that PERK/ATF4 signaling was involved in the ER stress-induced decrease in AR expression. To verify the functional role of ATF4 in ER stress-mediated decrease in AR expression, HCC2185, CAL-148, C4-2, and 22RV1 cells were transfected with the ATF4 overexpression plasmid. The results showed that AR levels were markedly reduced in cells transfected with the ATF4-overexpressing vector (Fig. 5d, e). Moreover, transfection with the ATF4-overexpressing vector decreased cell viability (Fig. 5f, g). Our results suggested that ATF4 overexpression inhibited AR in LAR TNBC and PCa cell lines. Therefore, PERK/ATF4 signaling contributed to ER stress-induced decrease in AR expression.

### ER stress promoted the binding of ATF4 to the AR promoter and inhibited AR promoter activity in LAR TNBC and PCa cell lines

**Fig. 6:**
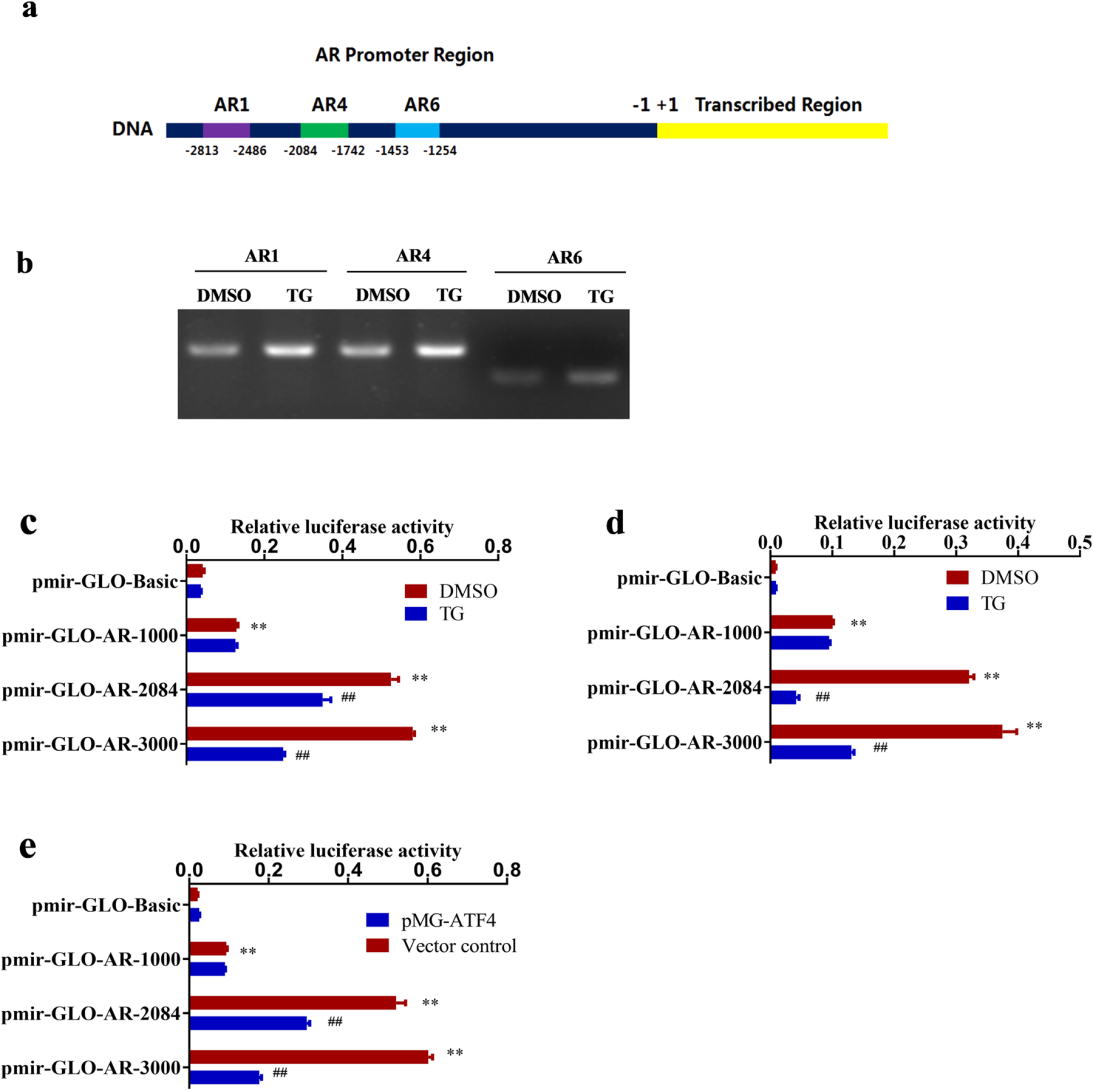
ATF4 bound to AR promoter region and inhibited AR promoter activity. **a** Schematic representation of the AR promoter and ATF4 binding sites. **b** MDA-MB-453 cells were treated with 1 μM TG for 24 h. Then ChIP-PCR was performed using anti-ATF4 antibodies. **c, d** MDA-MB-453 (**c**) and LNCap cells (**d**) were transfected with AR luciferase reporter plasmids or control plasmid pmir-Glo-Basic and then treated with 1 μM TG. Luciferase activities were measured using a dual-luciferase reporter assay system according to the manufacturer’s protocol. The relative luciferase activity was calculated and expressed as mean ± SD. Student’s *t*-test, **p < 0.01 *vs.* pmir-GLO-Basic (DMSO); ^##^*p* < 0.01 *vs.* corresponding pmir-GLO-AR (*n* = 3). **e** The AR luciferase reporter plasmids and ATF4-overexpressing plasmids or empty vector control were contransfected into MDA-MB-453 cells. Luciferase activities were measured. The relative luciferase activity was calculated and expressed as mean ± SD. Student’s *t*-test, **p < 0.01 *vs.* pmir-GLO-Basic (DMSO); ^##^*p* < 0.01 *vs.* corresponding pmir-GLO-AR (*n* = 3).

To further investigate the molecular mechanisms underlying the regulation of AR gene expression by ER stress, chromatin immunoprecipitation (ChIP)-PCR experiments were conducted. Chromatin DNA from MDA-MB-453 cells was extracted and purified after immunoprecipitation with anti-ATF4. The enriched DNA was detected using 11 primer pairs containing the AR promoter region utilizing PCR and then sequenced. The results showed that the PCR products (primer AR1: −2813–−2486 nt, primer AR4: −2084–−1742 nt, and primer AR6: −1453–−1254 nt) were found in the ATF4 immunoprecipitated group (Fig. 6a). In addition, the ChIP-PCR assay results also suggested that TG increased the binding of ATF4 protein to these three AR promoter regions (Fig. 6b). Moreover, the results of promoter and transcription factor binding site prediction analysis (http://jaspar.genereg.net/analysis) also showed that there were binding sites with a relative profile score threshold of >75% in the AR promoter region from −2813 to −2486 nt and from −2084 to −1742 nt. These results indicated that the nuclear transcription factor ATF4 could bind to the AR promoter region. Luciferase assays were performed to verify the binding of ATF4 to the AR promoter. MDA-MB-453 and LNCap cells were transfected with different reporters and then treated with or without TG. The results showed that in the absence of TG, the luciferase activities of the reporters pmir-GLO-AR-3000 and pmir-GLO-AR-2084 which contain AR gene regions from 0 to −3000 nt and 0 to −2084 nt, respectively, were significantly higher (*p* *<* 0.01) than those of the pmir-GLO-Basic (Fig. 6c, d), supporting the notion that these regions contained the AR promoter and/or cis-regulatory elements. TG decreased the luciferase activity of pmir-GLO-AR-3000 and pmir-GLO-AR-2084, but not pmir-GLO-AR-1000, in both MDA-MB-453 and LNCap cells (Fig. 6c, d), supporting the notion the above-identified AR promoter region from −2813 to −2486 nt and from −2084 to −1742 nt contains the cis-regulatory elements associated with ER stress. Similar results were observed when ATF4-overexpressing plasmids and AR luciferase reporter plasmids were co-transfected with MDA-MB-453 cells (Fig. 6e). The above results demonstrated that ATF4 could bind to the AR promoter region from −2813 to −2486 nt and from −2084 to −1742 nt, and that ER stress and ATF4 overexpression could inhibit AR promoter activity.

### AR and ATF4 displayed a negative correlation trend in breast cancers and PCas

**Fig. 7:**
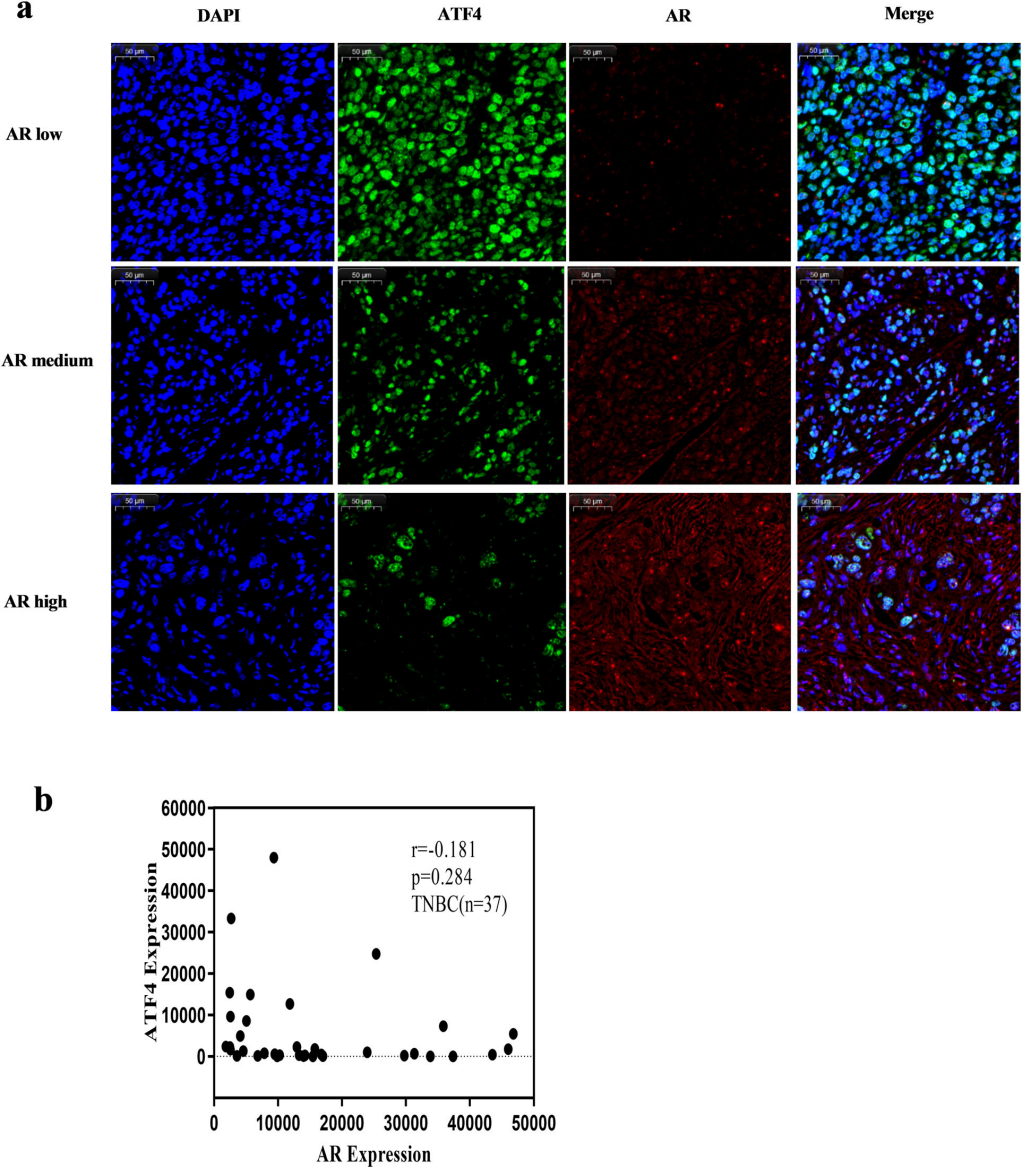
AR and ATF4 display a negative correlation trend in breast and prostate cancers. **a** The representative images of fluorescence expressions of AR and ATF4 in tissue microarray of TNBC tissues. Green represented ATF4, red represented AR. AR low: total fluorescence intensity was <12,500; AR medium: total fluorescence intensity was from 12,500 to 37,500; AR high: total fluorescence intensity was >37,500. scale bar: 50 μM. **b** The correlation of AR and ATF4 in tissue microarray of TNBC tissues (*n* = 37).

Using the Gene Expression Profiling Interactive Analysis (GEPIA) datasets (http://gepia.cancer-pku.cn/), we compared the mRNA expression of AR and ATF4 in clinical samples of breast cancers (*n* = 1085) and PCas (*n* = 492) available in the datasets. The results indicated that the expression levels of AR were higher in breast invasive carcinoma (BRCA) than in normal breast tissues, whereas the expression levels of ATF4 were lower in the former than in the latter (Supplementary Fig. 2a). Similar results were observed in prostate adenocarcinoma (PRAD) and normal prostate tissues (Supplementary Fig. 2b). AR and ATF4 displayed a negative correlation trend in BRCA (*r* = −0.22, Supplementary Fig. 2c) and PRAD (*r* = −0.19, Supplementary Fig. 2d). Furthermore, to determine whether AR expression was negatively correlated with ATF4 expression in TNBC, immunofluorescence double staining was used to detect the expression of AR and ATF4 in a TNBC tissue microarray. The results also showed a negative correlation trend between AR and ATF4 in TNBC (*r* = −0.181, Fig. 7a, b). However, as the number of tissue microarray cases was too small, the correlation between AR and ATF4 was not statistically different (*p* = 0.284, *n* = 37).

### ATF4 overexpression inhibited tumor growth in an orthotopic xenograft mouse model

**Fig. 8:**
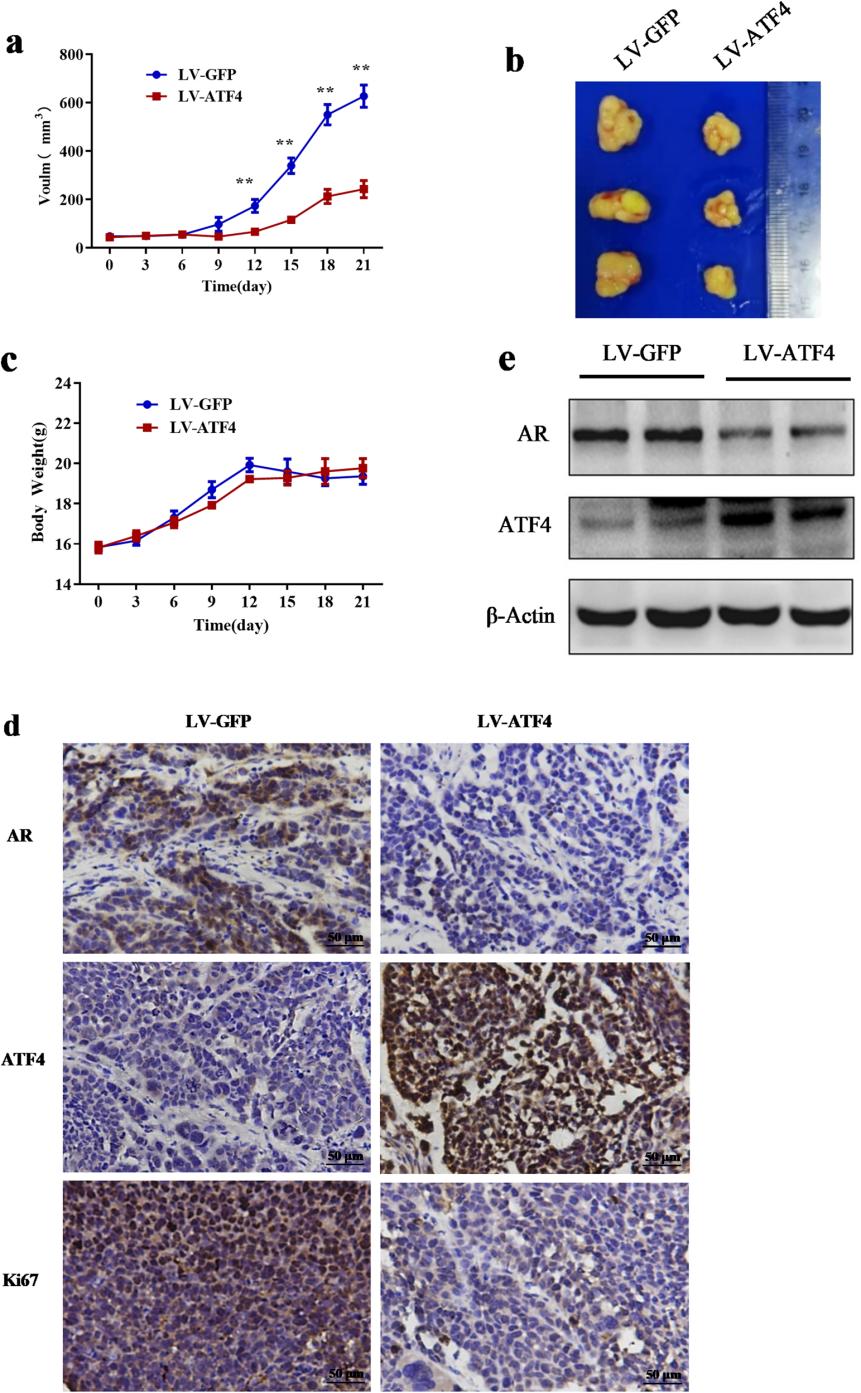
ATF4 overexpression inhibited tumor growth in a CAL-148 cell orthotopic xenograft mouse model. **a** Effect of ATF4 overexpression on tumor growth of CAL-148 xenografts. Tumor volume was measured every 3 days and expressed as mean ± SD. Student’s *t*-test, **p *<* 0.01 *vs*. LV-GFP, *n* = 5. **b** Representative image of tumors from each group. **c** Body weight changes in mice during the 21 days (*n* = 5). **d** Representative tumor tissues were sectioned and subjected to immunohistochemistry staining (magnification, ×400). scale bar: 50 μM. **e** Representative tumor tissues from each group were prepared and subjected to western blot assay.

To determine whether our findings could be replicated in vivo, CAL-148 cells stably overexpressing ATF4 lentivirus were orthotopically xenografted into nude mice. Tumor volumes were lower in mice with ATF4 overexpression xenografts than in control xenograft mice (Fig. 8a, b). Body weights did not differ among any of the groups of mice (Fig. 8c). Moreover, we analyzed the proliferative index in tumor sections and found that the expression levels of Ki-67, a marker of proliferation, and AR were much lower in ATF4-overexpressing xenograft mice than in the control xenograft mice (Fig. 8d). Western blotting also verified that ATF4 overexpression decreased AR expression (Fig. 8e). Taken together, these results suggested that ATF4 overexpression exerted an antitumor effect through the ATF4 pathway by inhibiting AR expression.

## Discussion

**Fig. 9:**
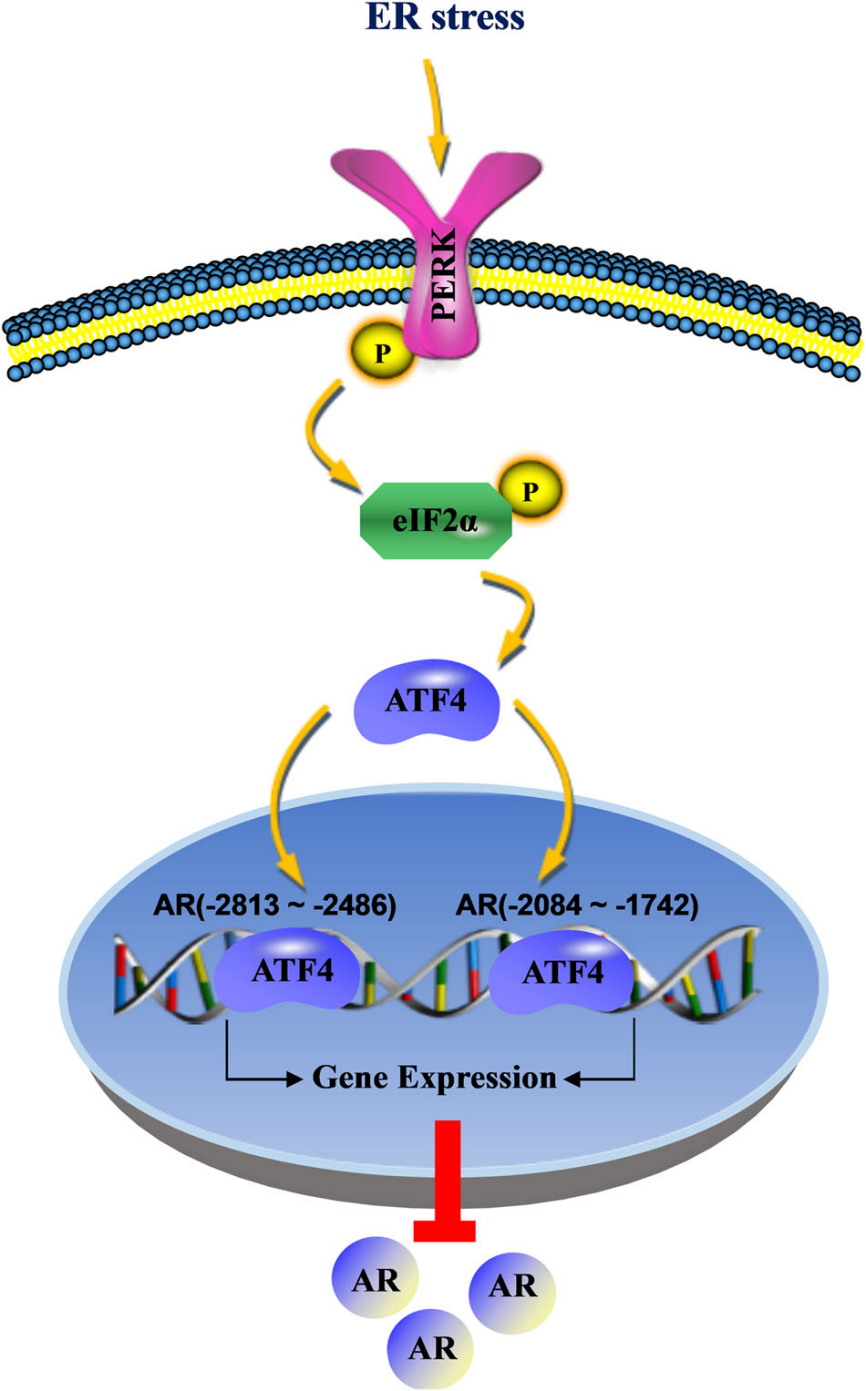
A proposed model for ER-stress-induced AR decrease. ER-stress activates PERK/ATF4 signaling pathway, then nuclear transcription factor ATF4 binds to the AR promoter region to inhibit AR promoter activity, resulting in suppression of AR expression. Endoplasmic reticulum is abbreviated as ER.

In this study, we demonstrated that ER stress decreased AR expression at the transcriptional level via PERK/eIF2α/ATF4 signaling in LAR TNBC and PCa. In addition, we identified that ATF4 binds to the AR promoter region from −2813 to −2486 nt and from −2084 to −1742 nt to inhibit AR promoter activity (Fig. 9). Targeting ER stress could be an effective strategy for the treatment of AR-dependent TNBC and PCa.

PCa is the most common cancer and the leading cause of cancer-related deaths in men worldwide^4,18,19^. Although the current primary treatment for PCa remains androgen-deprivation therapy (ADT), the duration of response varies from months to years and the disease inevitably progresses to a lethal stage called castration-resistant PCa (CRPC)^20,21^. Although second-generation AR antagonists, such as abiraterone and enzalutamide, are both approved for the treatment of CRPC, the inherent or acquired resistance of CRPC to these drugs remains a major clinical obstacle^4,22–25^. Therefore, the development of novel agents targeting the AR pathway is urgently needed, and downregulation of AR expression is a promising alternative treatment strategy.

Some studies have reported that androgens mainly inhibit tumor growth in ERα-positive breast cancer^26^. However, in ERα-negative/AR-positive cells, they accelerate tumor growth^27,28^. In a phase II study, androgen deprivation by abiraterone acetate combined with prednisolone had some responses in AR-positive/metastatic TNBC^29,30^. Preclinical and clinical data showed that targeted inhibition of AR could be a potential therapy for AR-positive/ER-negative breast cancer patients. LAR TNBC is an ERα-negative/AR-positive breast cancer and anti-androgens may be effective in its management. Therefore, AR-targeted therapies require further evaluation for LAR TNBC.

The AR signaling cascade can be inhibited by several treatment approaches^20,21^. AR signaling can be indirectly inhibited by decreasing circulating androgen levels through ADT.^25^ Moreover, AR expression can be directly blocked by treatment with AR antagonists, such as bicalutamide and enzalutamide^31^. However, some studies have reported that there are many problems with the direct or indirect methods associated with the resistance to AR-targeted therapies in PCa because of amplification or overexpression of AR, ligand-binding domain mutations, ligand-independent activation, and the expression of active AR splice variants. Therefore, promoting AR degradation or inhibiting AR synthesis may serve as a novel strategy for inhibiting AR signaling^32,33^.

ER stress triggers the UPR, which results in a temporary blockade of global mRNA translation to alleviate the accumulation of misfolded proteins in the ER^34^. ER stress may activate the UPR to regulate associated protein expression and may be closely related to tumor growth and drug resistance. Some studies have shown that ER stress plays a role in regulating AR signaling. For example, riluzole induces AR protein degradation via ER stress pathways to exert its anti-PCa effects^35^; rosemary extracts modulate ER stress to promote AR degradation in PCa^36^; TG analogs containing amino acids deplete AR protein via synthesis inhibition and induce the death of PCa cells^37^. However, the detailed mechanisms by which ER stress regulates AR response in PCa are still not fully understood, and the relationship between ER stress and AR expression in LAR TNBC remains unknown. Our results showed that treatment with different ER stress inducers (i.e., TG and BFA) significantly decreased the AR levels in LAR TNBC and PCa cell lines. In addition, TG and BFA decreased AR mRNA and protein levels in LAR TNBC and PCa cell lines. Furthermore, we found that ER stress did not promote AR mRNA and protein degradation based on the results of the experiments using the protein synthesis inhibitor CHX and the general transcription inhibitor ActD. These results indicate that ER stress inhibits AR transcription and reduces AR expression.

Our results indicate that the PERK pathway, but not the IRE1α and ATF6 pathways, participates in the ER stress-induced AR decrease. Further mechanistic investigations demonstrated that ATF4 can bind to the AR promoter region from −2813 to −2486 nt and from −2084 to −1742 nt, and that TG and ATF4 overexpression inhibited AR promoter activity. Therefore, we conclude that the PERK/eIF2α/ATF4 pathway is responsible for the ER stress-induced decrease in AR expression.

In our study, an orthotopic xenograft mouse model was used to evaluate AR expression and its inhibitory effects on tumor growth in vivo by lentivirus-mediated delivery of ATF4. Our results showed that overexpression of ATF4 inhibited tumor growth and AR expression in a CAL-148 cell xenograft mouse model. The above results establish that ER stress inhibits AR expression via the PERK/eIF2α/ATF4 pathway activation. The in vivo results were consistent with those obtained in vitro.

In conclusion, our findings demonstrate that AR is a new ER stress response gene and that the PERK/eIF2α/ATF4 signaling pathway participates in this response. Our findings may lead to a better understanding of ER stress activation and AR expression control in AR-dependent TNBC and PCa. Targeting UPR may be a strategy for the treatment of AR-dependent TNBC and PCa.

## Methods

### Materials

TG, BFA, TM, 4μ8c, GSK2656157, CHX, and ActD were purchased from MedChemExpress (Monmouth, NJ, USA). Female nude mice (5 weeks old, weight 18–22 g) were obtained from Beijing HFK Bioscience Co.,Ltd. (Beijing, China). TNBC tissue microarray was obtained from Avilabio Biotechnology Co., Ltd. (Shanxi, China). Matrigel was purchased from BD Biosciences (San Jose, CA, USA). Antibodies against PERK (Cat#5683), eIF2α (Cat#5324), p-eIF2α (Cat#3398), ATF4 (Cat#11815), ATF6 (Cat#65880), IRE1α (Cat#3294), CHOP (Cat#2895), XBP1s (Cat#12782), Bip (Cat#3177) and AR (Cat#5153) were obtained from Cell Signaling Technology (Danvers, MA, USA). The Ki-67 antibody (Cat#AF0198) was obtained from Affinity Biosciences (Zhenjiang, China), and the β-actin antibody was obtained from Santa Cruz Biotechnology (Santa Cruz, CA, USA). Cell Counting Kit-8 (CCK-8) was purchased from Bimake (Houston, TX, USA). RIPA lysis buffer, BCA protein assay kit, and dual-luciferase reporter assay kit were obtained from Beyotime (Shanghai, China). ChIP assay kit was purchased from Thermo Fisher Scientific (Waltham, MA, USA). PERK siRNA, ATF4 siRNA, CHOP siRNA, and control siRNA were purchased from Santa Cruz Biotechnology (Santa Cruz, CA, USA); ATF6 siRNA was obtained from RiboBio (Guangzhou, China); IRE1α siRNA, IRE1α-overexpressing lentivirus (LV-IRE1α), ATF4-overexpressing lentivirus (LV-ATF4) and negative control lentivirus (LV-GFP) were purchased from GeneChem (Shanghai, China).

### Cell culture

Human breast cancer cell lines (MDA-MB-453, CAL-148, HCC2185, and MFM-223) and human PCa cell lines (LNCap, C4-2, and 22RV1) were obtained from the American Type Culture Collection. MDA-MB-453 cells were cultured in Leibovitz’s L-15 medium and the other cell lines were cultured in DMEM-high glucose medium supplemented with 10% fetal bovine serum. Cells were grown at 37 °C within a humidified atmosphere containing 5% CO_2_. All the cells were verified for mycoplasma contamination free.

### CCK-8 assay

CCK-8 assay was used to measure cell viability. Cells were seeded in a 96-well plate. Then, the cells were directly treated with 10 or 12 μM CCT020312 or 1 μM TG for 24 h. Subsequently, 10 µL of CCK-8 solution was added to each well, and the cells were subsequently incubated for 1 or 2 h at 37 °C. Absorbance was measured at 450 nm using a full-wavelength microplate reader (Thermo Fisher Scientific).

### RNA preparation and quantitative RT-PCR (RT-qPCR)

Total RNA was extracted from cells using the TRIzol reagent. Next, 2 μg of total RNA was reverse-transcribed into cDNA using Superscript III (Invitrogen) following the manufacturer’s instructions. qPCR was performed using SYBR Premix Ex Taq^TM^ II Kit. The primers used for qPCR are listed in Supplementary Table 1. RT-qPCR was performed using an iCycler Thermal Cycler (Bio-Rad) under the following reaction conditions: denaturation at 94 °C for 300 s (1 cycle); denaturation at 94 °C for 30 s, annealing at 60 °C for 30 s, and extension at 72 °C for 45 s (40 cycles). A dissociation curve analysis was performed to determine whether there were any bimodal dissociation curves or abnormal amplification plots. For each sample, mRNA expression levels for specific transcripts were normalized to the expression of β-actin, and the 2^−ΔΔ^CT method was used to analyze the gene expression data.

### Western blotting analysis

Total cell protein was extracted using the RIPA lysis buffer, and the protein concentration was measured using a BCA Protein Assay Kit. Subsequently, the equivalent amount of proteins was separated using SDS-polyacrylamide gel electrophoresis and transferred to polyvinylidene fluoride (PVDF) membranes. Immunoblotting was performed as previously described^38^. Briefly, the transferred PVDF membranes were blocked by incubation with 5% non-fat milk in TBST buffer for 1 h, and then incubated with primary antibody (diluted in 5% non-fat milk or 5% BSA in TBST buffer) at 4 °C overnight. The dilution for the primary antibodies was 1:1000 unless otherwise indicated. After washing four times with TBST buffer, the membranes were incubated with the HRP-conjugated secondary antibodies for 1 h. Then, the membranes were washed four times and incubated with the enhanced chemiluminescence (ECL) substrate. The signals on the membranes were captured by the ChemiDoc MP imaging system. All blots derived from the same experiment and were processed in parallel.

### Immunohistochemistry staining

Tumor specimens were embedded in paraffin and cut into 5 μm-thick sections. Samples were incubated with a specific primary antibody at 4 °C overnight and incubated with HRP-conjugated secondary antibodies at 25 °C for 2 h. The signal was visualized with DAB reagent and examined under a light microscope.

### Immunofluorescence staining

The TNBC tissue microarray was incubated with a specific primary antibody at 4 °C overnight. The cells were then incubated for 1 h with Alexa Fluor 488-conjugated goat anti-mouse and Alexa Fluor 647-conjugated donkey anti-rabbit secondary antibodies (Molecular Probes). Nuclei were counterstained with 0.1 μg/mL DAPI (Sigma). Total fluorescence intensity was examined and analyzed using the Pannoramic MIDI scanner from “3DHISTECH”.

### ChIP -PCR assay

Cells (3 × 10^6^/mL) were seeded in 10 cm cell culture dishes and treated the following day with 1 μM TG for 24 h. The ChIP assay was performed according to the manufacturer’s instructions (Thermo Fisher Scientific). Anti-ATF4 antibodies were used for immunoprecipitation, and normal mouse IgG was used as a control in ChIP analysis. DNA was amplified using PCR with a TaKaRa Ex Taq DNA polymerase kit. PCR has performed under the following reaction conditions: denaturation at 94 °C for 30 s, annealing at 58 °C for 30 s, and extension at 72 °C for 60 s (30 cycles). The PCR products were identified using 1.0% agarose gel electrophoresis and ethidium bromide staining. Images of the gels were obtained using a Tanon 3200 Gel Imaging System. The primers used for the PCR are listed in Supplementary Table 2.

### Plasmid construction

To generate the ATF4-overexpressing plasmid (pMG-ATF4), the DNA fragment encoding ATF4 was generated using PCR with RNA from MCF7 cells using the following primers: 5′-CTGAGATCTCACCATGACCGAAATGAGCTTCC-3′ and 5′-GGGGCTAGCCTAGGGGACCCTTTTCTTC-3′. The coding sequence of ATF4 was cloned into the mammalian expression vector pMG-H2 using *Bgl*II-*Nhe*I restriction sites. The AR-overexpressing plasmid was a gift from Dr. Yuanzhong Wang from the Beckman Research Institute of the City of Hope. All inserts were verified using DNA sequencing.

### Transient transfection and luciferase reporter assay

Cells were seeded in 24-well plates at a density of 5 × 10^4^ cells/well for 24 h. The cells in each well were transfected with 1 μL of Lipofectamine 2000 and 0.2 μg of reporter plasmids in 50 μL of Opti-MEM. Six hours after transfection, 500 μL of cell culture medium was added to each well. After 48 h of incubation, the cells were washed once with PBS. A luciferase reporter assay was performed using a dual-luciferase reporter assay system according to the manufacturer’s protocol. The relative luciferase activity was calculated. The data are expressed as the means ± SD.

### Cell transfection and transduction

Cells were transfected with siRNA or plasmids using RNAiMax or Lipofectamine 3000 and Opti-MEM I reduced serum medium for 6–12 h. Then, the cells were replaced with the normal growth medium and subjected to further treatment as indicated.

For lentivirus transduction, cells were cultured to 50% confluence and transduced with IRE1α lentivirus (LV-IRE1α), ATF4 lentivirus (LV-ATF4), or control lentivirus (LV-GFP) according to the protocol in the Lentivirus Operation Manual. The culture was renewed in a regular medium for 72 h, and the cells were subjected to further treatment.

### GEPIA dataset

Gene Expression Profiling Interactive Analysis (GEPIA, http://gepia.cancer-pku.cn) is a newly developed interactive web server for analyzing the RNA sequencing expression data of 9736 tumors and 8587 normal samples from TCGA and GTEx projects, using a standard processing pipeline. GEPIA provides customizable functions such as tumor/normal differential expression analysis, profiling according to cancer type or pathological stage, patient survival analysis, similar gene detection, correlation analysis, and dimensionality reduction analysis^39^. For analyzing relative expression of AR or ATF4 between breast tumors and normal breast tissues or between PCa and normal prostate tissue, Single Gene Analysis under GEPIA (http://gepia.cancer-pku.cn) was chosen. After entering the analyzed gene name (ATF4 or AR), “Boxplot” was clicked, and BRCA (breast invasive carcinoma) or PRAD (prostate adenocarcinoma) was selected as the datasets for analysis accordingly. We kept the others as the default setting (|Log2FC| Cutoff: 1; *p*-value Cutoff: 0.01); log scale: Yes; Jitter Size: 0.4; Match TCGA normal and GTEx data. After clicking “Plot”, the plot box results of relative expression of AR or ATF4 between breast tumors and normal breast tissues or between PCa and prostate normal tissue were generated. For generating expression correlations between AR and ATF4 in breast cancer and in PCa, Multiple Gene Analysis under GEPIA was chosen and Correlation Analysis was selected. After entering “AR” as Gene A and “ATF4” as Gene B, we chose “Pearson” as Correlation Coefficient and selected “BRCA Tumor” or “PRAD” under TCGA Tumor as the analysis datasets. After clicking “Plot”, the plot results for correlation analysis results were generated. During the whole analysis, no accession numbers or codes are needed or generated.

### Orthotopic implantation model and treatment

Female nude 5 weeks old mice weighing 18–22 g were obtained from Beijing HFK Bioscience Co., Ltd. (Beijing, China) and housed in a pathogen-free facility with a 12 h artificial light–dark cycle in Chongqing Medical University. The experiments were performed in accordance with the National Guidelines for Animal Care and Use and approved by the Animal Care and Use Committee of Chongqing Medical University. An orthotopic implantation model at the eighth breast with CAL-148 cells (5 × 10^6^ cells/0.05 mL) stably expressing LV-GFP or LV-ATF4 mixed with Matrigel (1:1) was established in nude mice (five mice/group). When tumors grew to ~40–50 mm^3^, tumor growth in the mice was measured every third day. The tumor volume (*V*) was measured using a slide caliper and calculated using the following formula: *V* (mm^3^) = 0.5 × ab^2^, where *a* and *b* represent the long diameter and perpendicular short diameter (mm) of the tumor, respectively.

### Statistical analyses

The data were analyzed using GraphPad Prism 8.0 software (GraphPad, Inc., Chicago, IL, USA). All data are expressed as mean ± SD. The student’s *t*-test was used to compare two values (two-tailed, two-sample equal variance). Comparisons among multiple groups were performed using one-way ANOVA. The criterion for statistical significance was set at *p* < 0.05.

### Reporting summary

Further information on research design is available in the Nature Research Reporting Summary linked to this article.

## Supplementary information

REPORTING SUMMARY

Supplementary

## Data Availability

All uncropped western blot generated during this study are available in supplementary Fig. 3. Plasmid pMG-ATF4 was deposited in Addgene, with ID number 80445. The data presented in this article are available from the corresponding author upon request.
