# Supplementary material for: Endoplasmic reticulum stress inhibits AR expression via the PERK/eIF2α/ATF4 pathway in luminal androgen receptor triple-negative breast cancer and prostate cancer
Source: NPJ Breast Cancer. 2022 Jan 10;8:2. doi: 10.1038/s41523-021-00370-1 (PMC8748692; doi:10.1038/s41523-021-00370-1)

Supplementary Table 1. The RT-PCR primers

Gene		Primer
β-actin	Forward	ACCCCGTGCTGCTGACCGAG
	Reverse	TCCCGGCCAGCCAGGTCCA
BiP	Forward	TGTCGCCTTCACTCCTG
	Reverse	ACAGACGGGTCATTCCAC
CHOP	Forward	GCCACTCCCCATTATCCT
	Reverse	CCTCATACCAGGCTTCCA
AR	Forward	CCATCCACGTTGTCCCT
	Reverse	GCTGTTGCTGAAGGAGTTG

Supplementary Table 2 The RT-PCR primers

Gene		Primer
AR1	Forward	AGGTGGAGGGAAAATGTACAAAGTG
	Reverse	CTGCCCATTTATTTCATCTCTGTTG
AR2	Forward	TGGCAACAACAGAGATGAAATAAAT
	Reverse	TTTGTACCAGCTTTCTTCTTCTTTC
AR3	Forward	TGCAAAGAACAGGAGGAGAAAGAAG
	Reverse	CAGAAGGCTAGTGTGTCATTTAAAAGA
AR4	Forward	AATTGTTGACAGCACCATCTTTAAA
	Reverse	ATGAACAATGGACAGGAGTGAAGAG
AR5	Forward	TCCTCTTCACTCCTGTCCATTGTTC
	Reverse	GGCTATAAATGAACCTTCCAGAACA
AR6	Forward	TTTGTGTTCTGGAAGGTTCATTTAT
	Reverse	CTAAAGGCAAGGAAGACTGGATATG
AR7	Forward	GCCTATCATATCCAGTCTTCCTTGC
	Reverse	CTGTGTAAAGGTGGAGATGCAAGTG
AR8	Forward	CCCACTTGCATCTCCACCTTTACAC
	Reverse	AAGGCAAAATCACCCAGACAAGCAG
AR9	Forward	GTTTGTGTGTTTACCTGCTTGTCTG
	Reverse	AGAGCGCGCAGATGGGAGAGT
AR10	Forward	TCATGGCTTGCTCCTCAGTTTGTA
	Reverse	GGCGAGGGCAGGAGAGGCTAG
AR11	Forward	TCTGGAGCCCTGGCGCCTAAA
	Reverse	AGGGGGCGCTGGGAGGTGGA

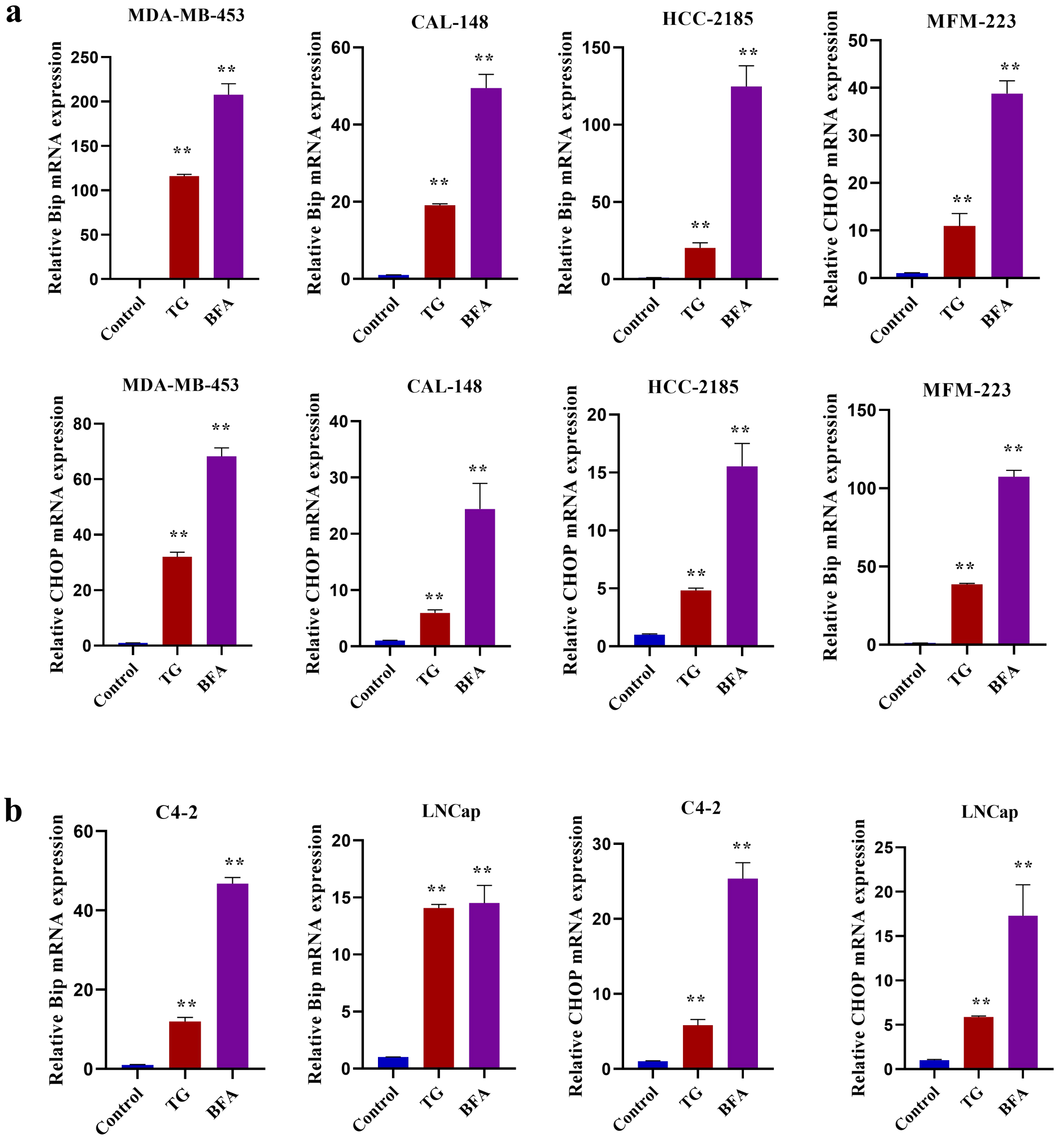

Supplementary Figure 1. ER-stress up-regulated Bip and CHOP mRNA expressions in LAR TNBC and PCa cells. **a, b** Breast cancer cell lines MDA-MB-453, CAL-148, HCC2185 and MFM-223 (a), and prostate cancer cell lines C4-2 and LNCap cells (b) were treated with 1 μM TG or 5 μg/mL BFA for 24 h. Then the cells were collected for quantitative RT-PCR assay. The relative expression of each target was normalized to β-actin and expressed as mean ± SD. Student's t-test, ***p*<0.01 *vs.* Control, n=3. Thapsigargin and Brefeldin A is abbreviated as TG and BFA, respectively.

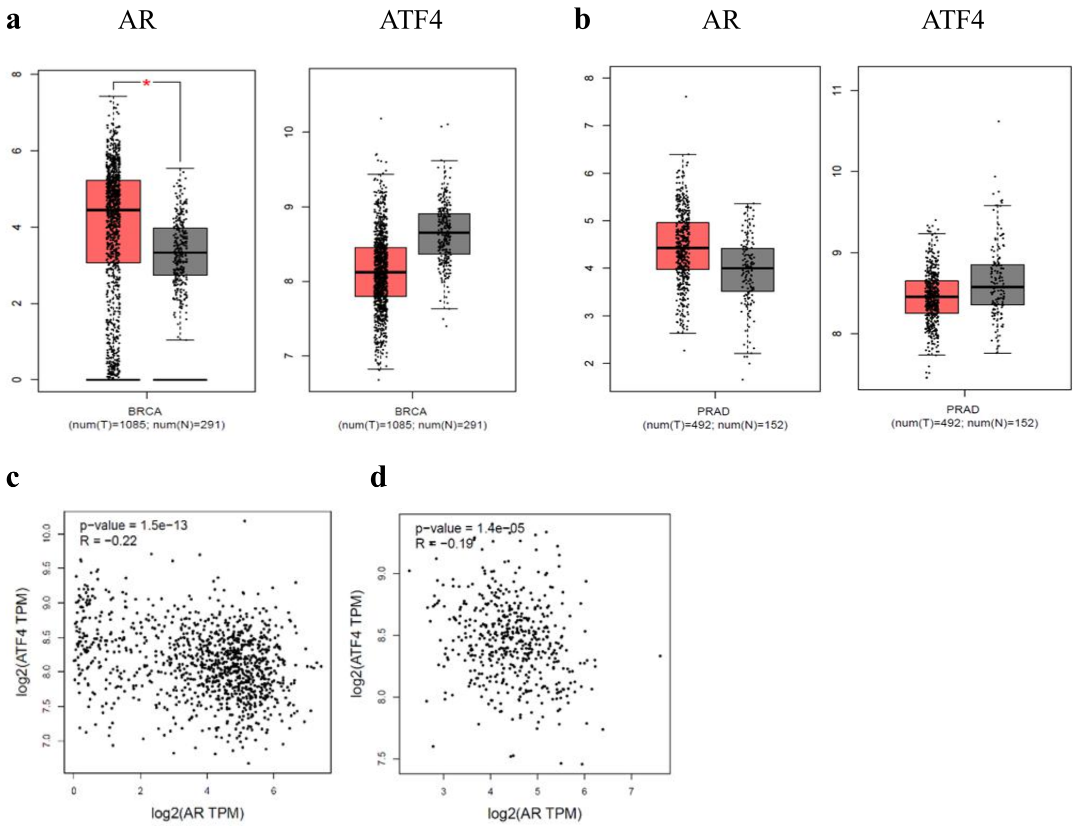

Supplementary Figure 2. The relative AR and ATF4 mRNA expression in breast cancer and prostate cancer. a, b The relative mRNA expression of AR and ATF4 between breast tumors and normal breast tissues (a) and between prostate tumors and normal prostate tissues (b) in GEPIA dataset. *p<0.05. Study the correlation of AR and ATF4 in breast cancer (d) and prostate cancer (d) in GEPIA dataset.

Supplementary Figure 3

Figure 1 a

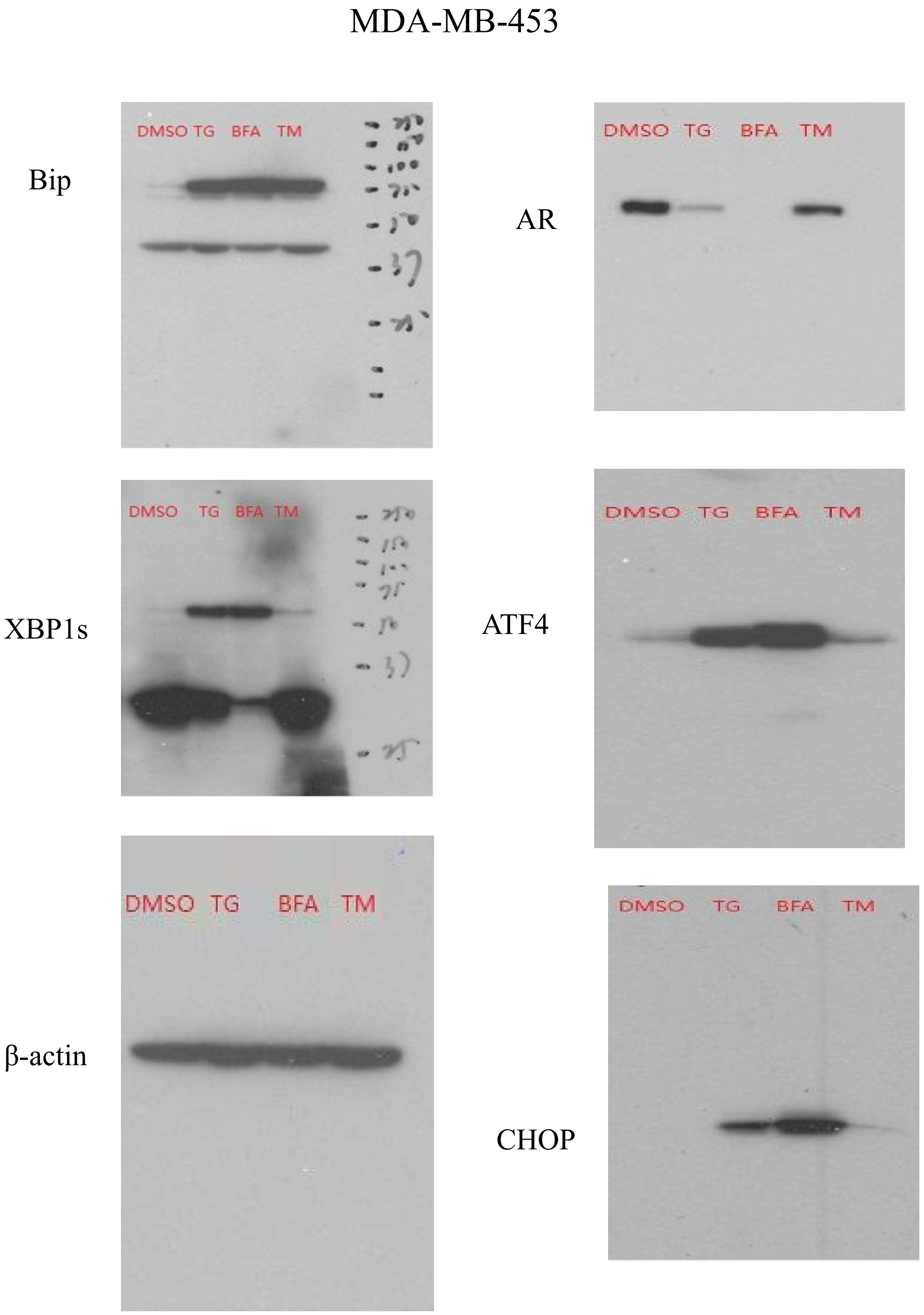

Figure 1 a

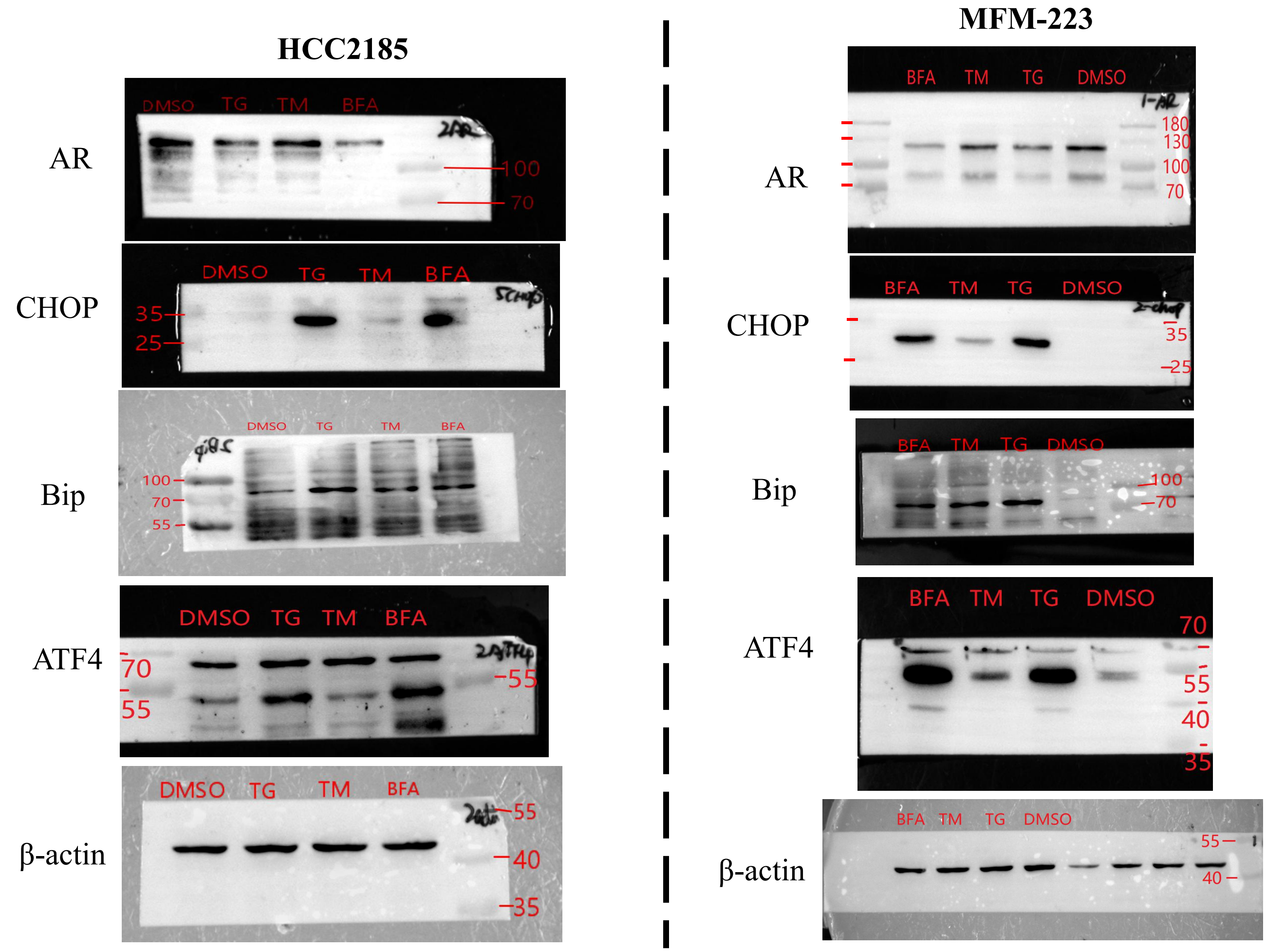

Figure1 b

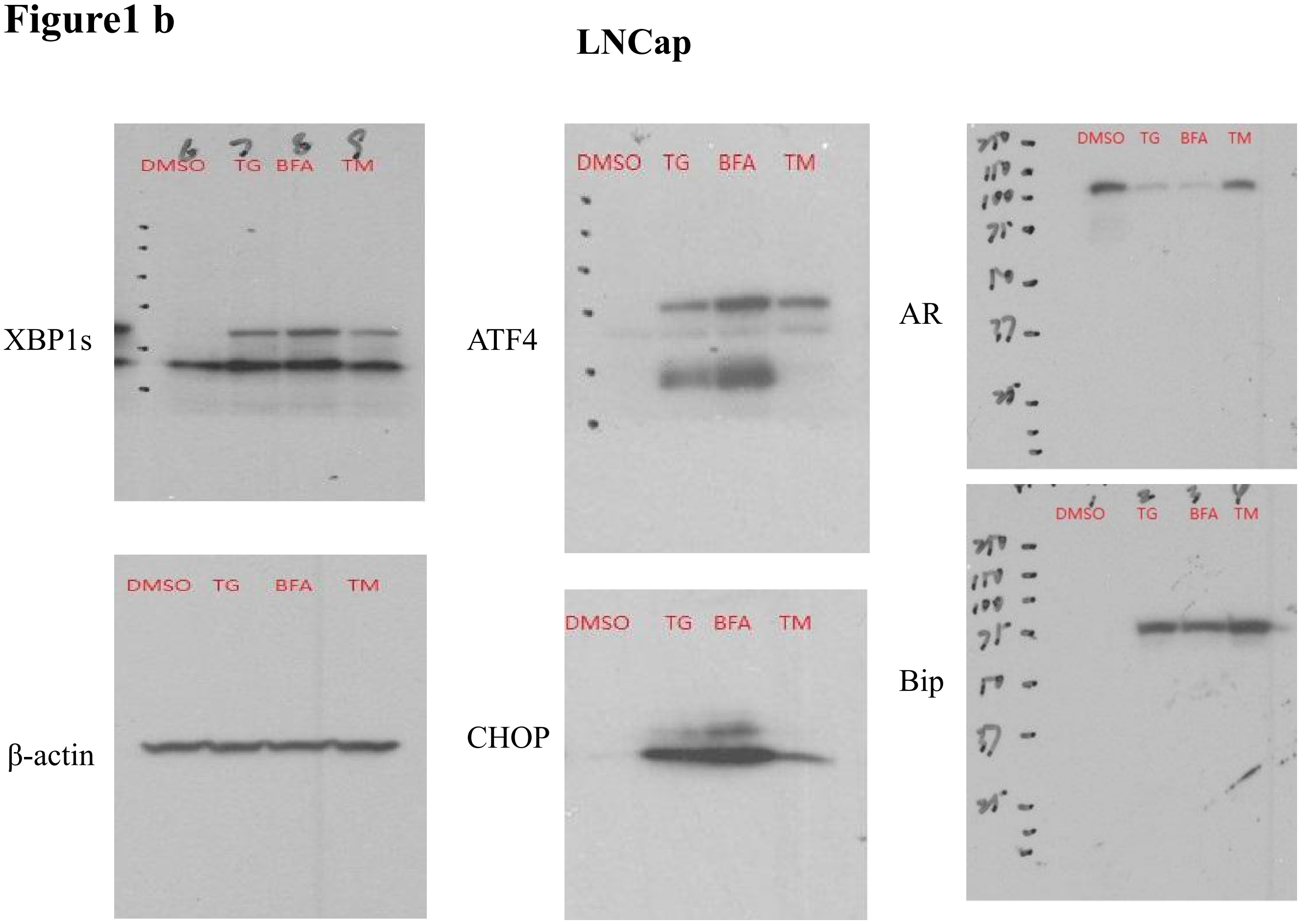

Figure1 b

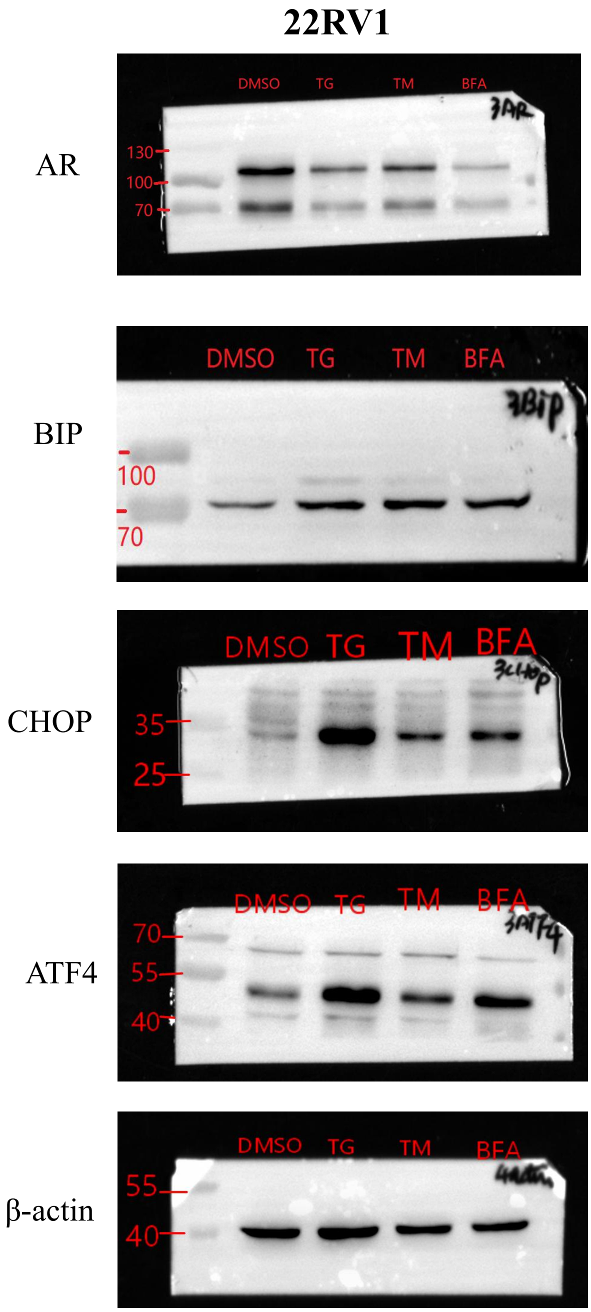
22RV1
AR
DMSO TG TM BFA
130
100
70
BIP
DMSO TG TM BFA
100
70
CHOP
DMSO TG TM BFA
35
25
ATF4
DMSO TG TM BFA
70
55
40
β-actin
DMSO TG TM BFA
55
40

Figure1 c

MDA-MB-453

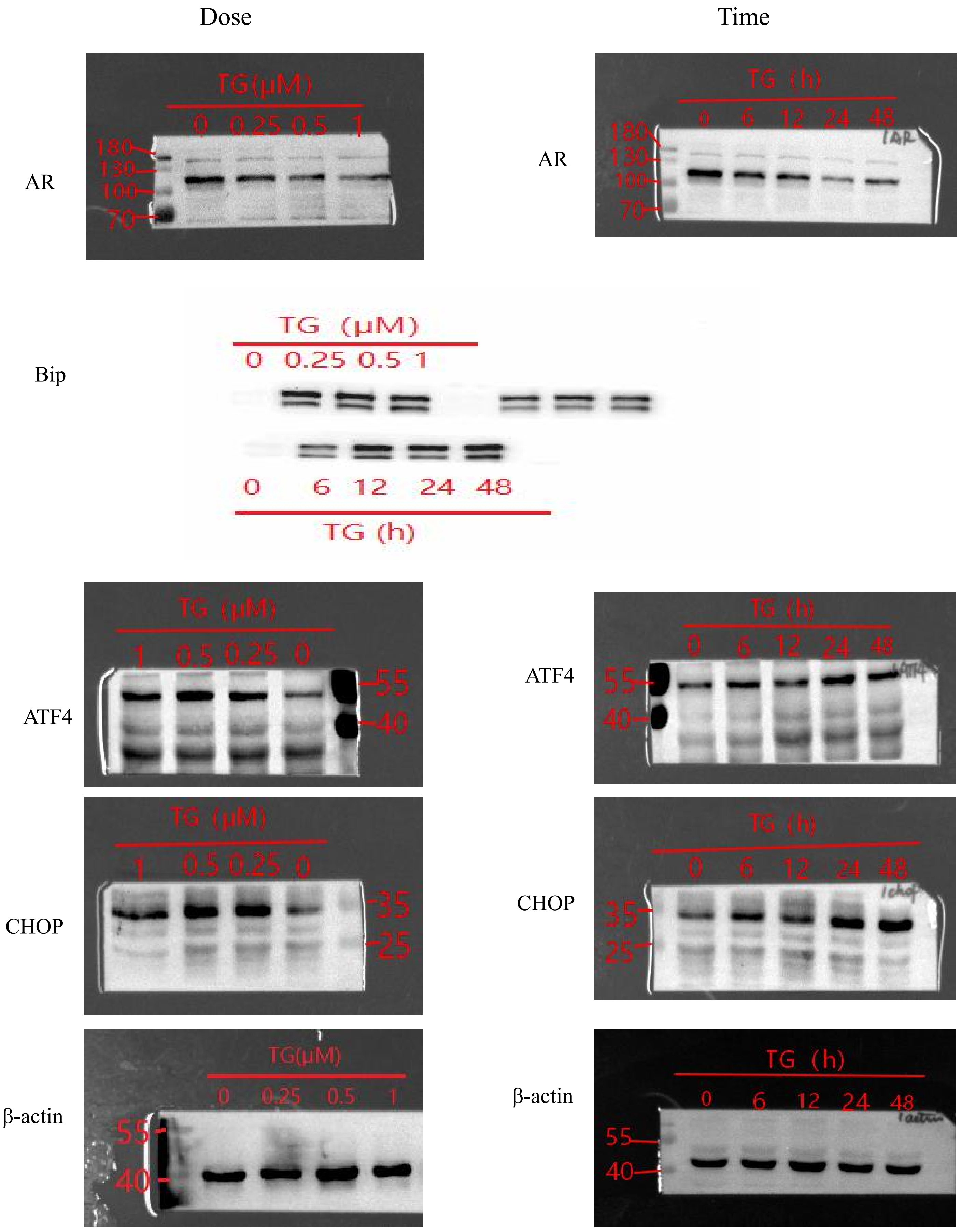

Figure1 c

CAL-148

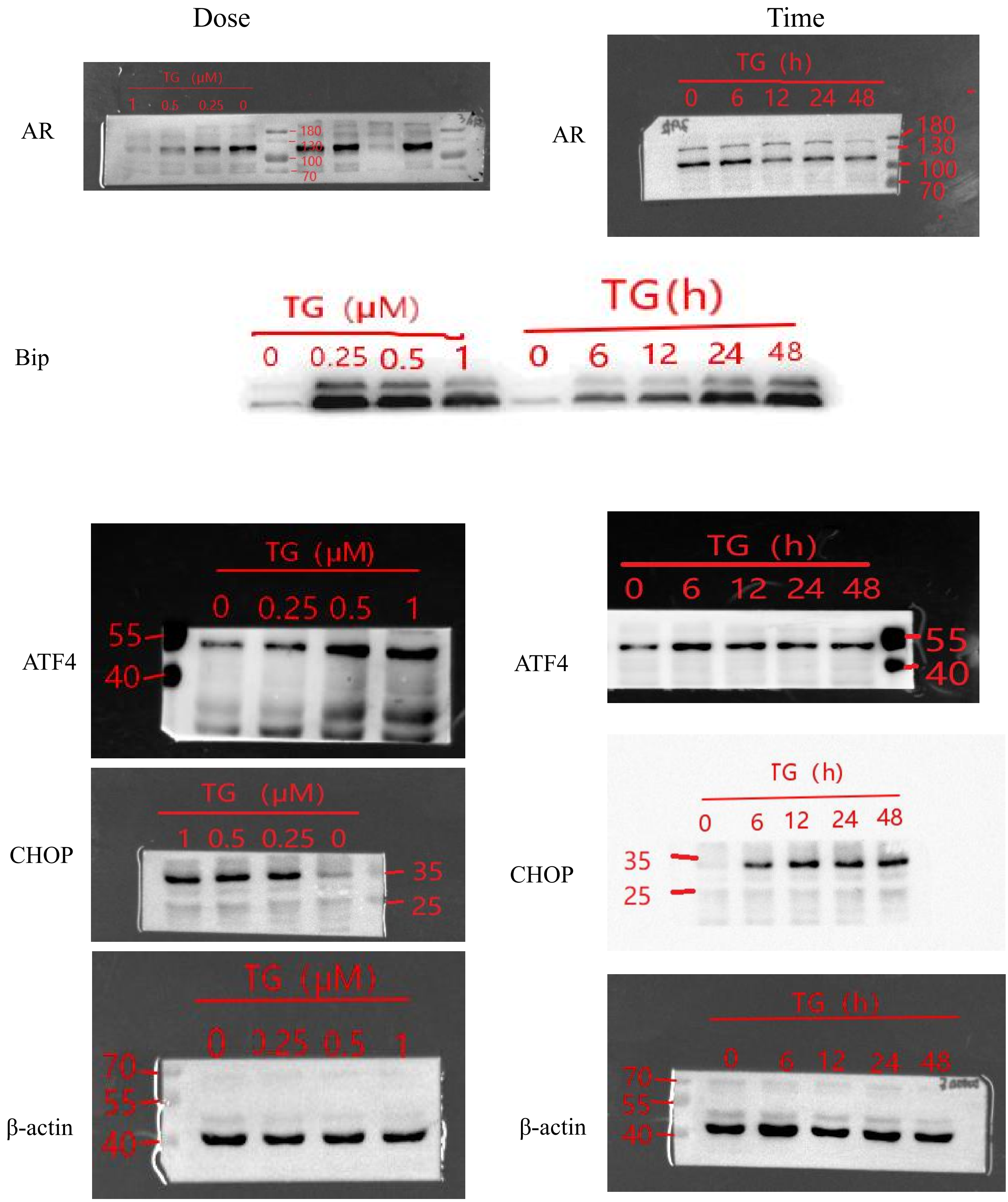

Figure1 d

LNCap

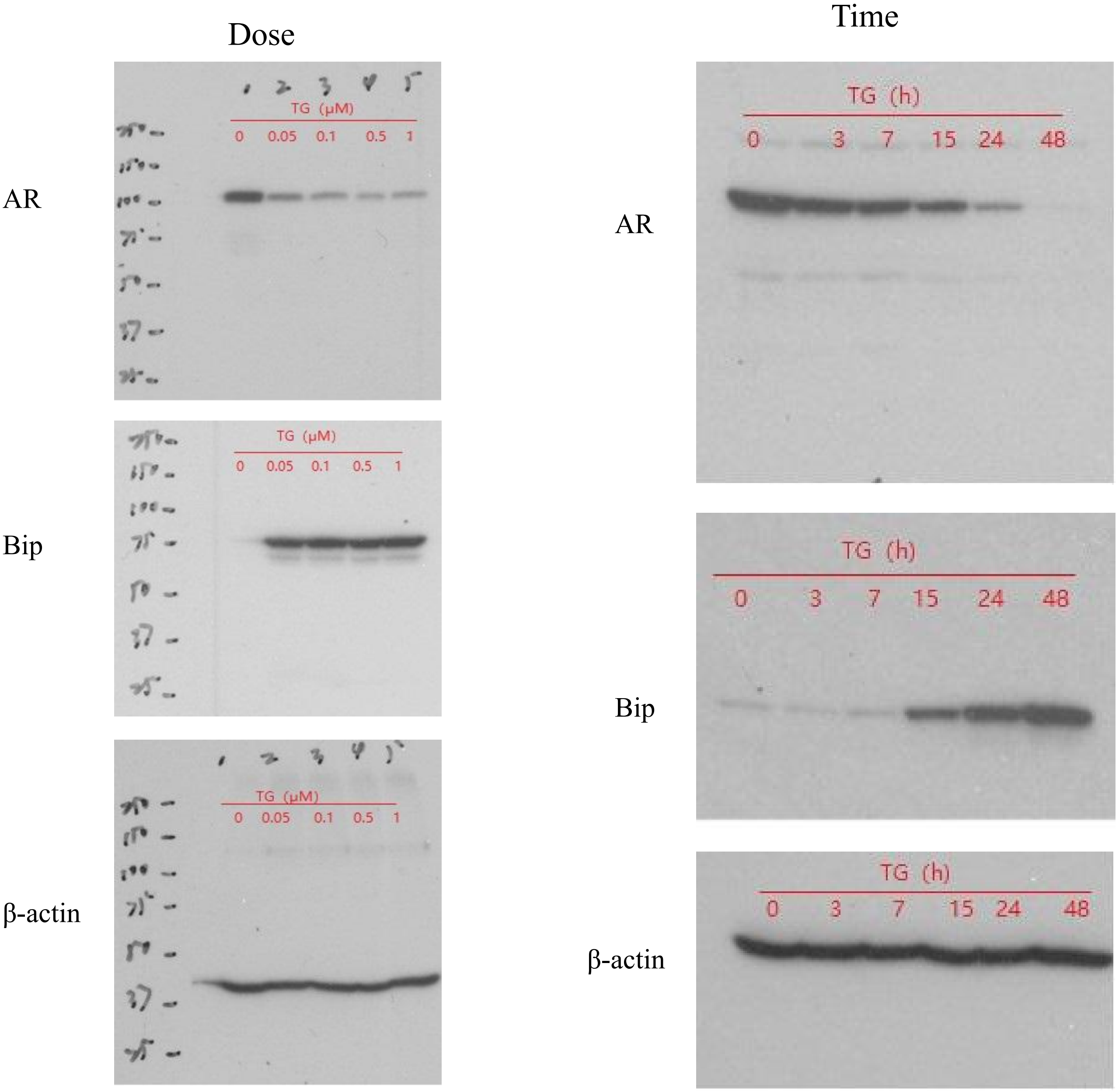

Figure1 d

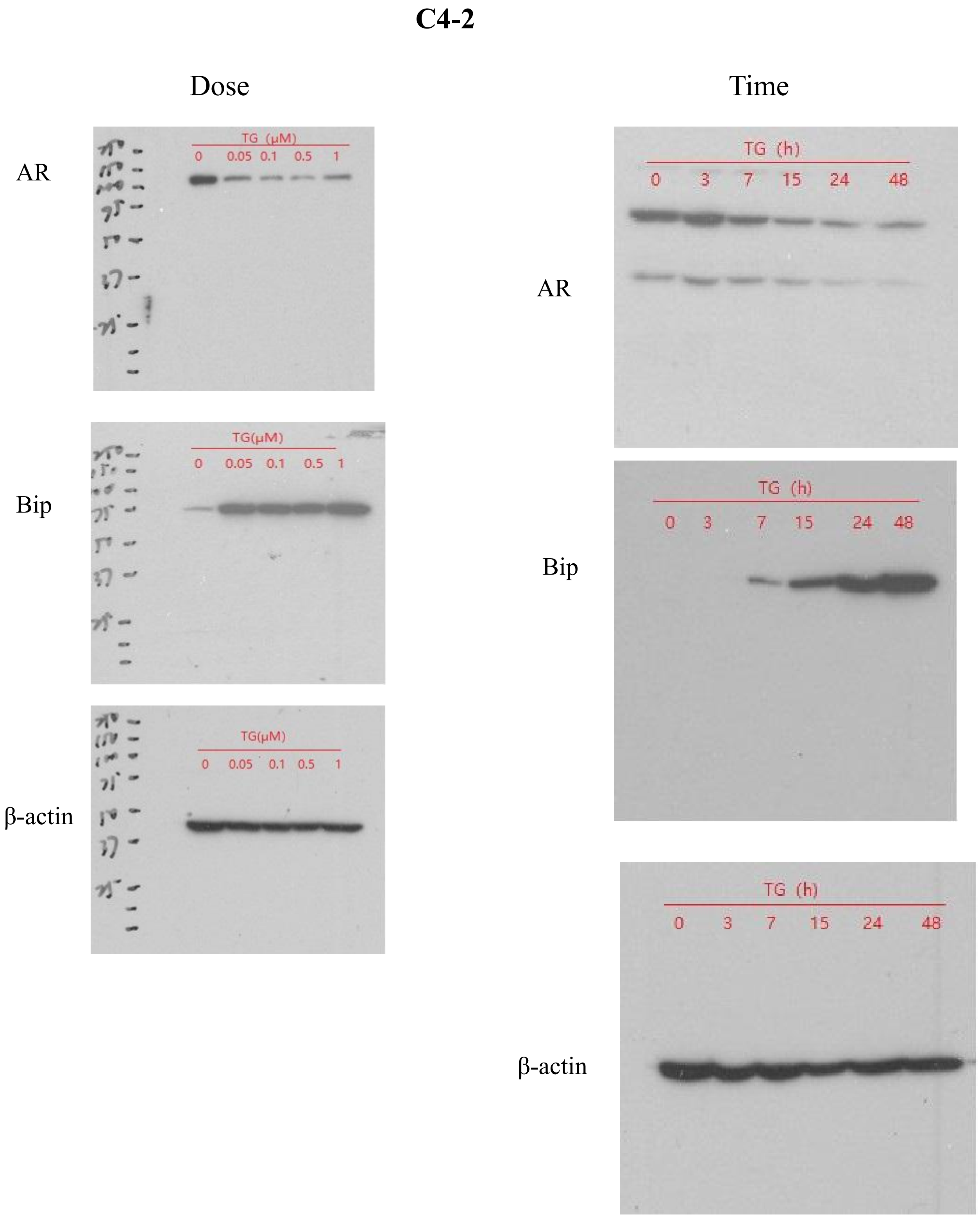

C4-2
Dose
Time
AR
TG (μM)
0 0.05 0.1 0.5 1
Bip
TG(μM)
0 0.05 0.1 0.5 1
β-actin
TG(μM)
0 0.05 0.1 0.5 1
TG (h)
0 3 7 15 24 48
AR
TG (h)
0 3 7 15 24 48
Bip
TG (h)
0 3 7 15 24 48
β-actin

Figure2 c

MDA-MB-453

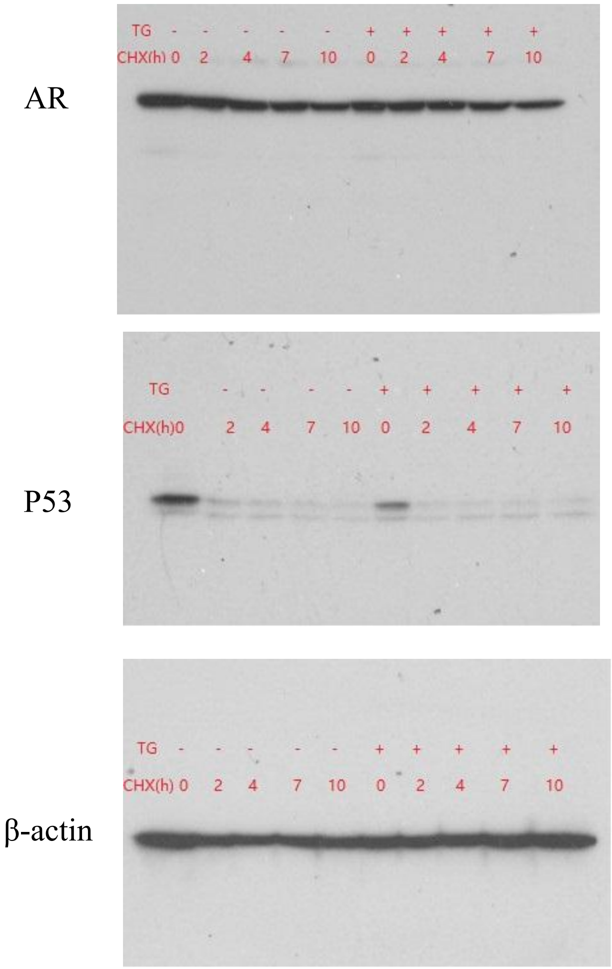

Figure2 d

CAL-148

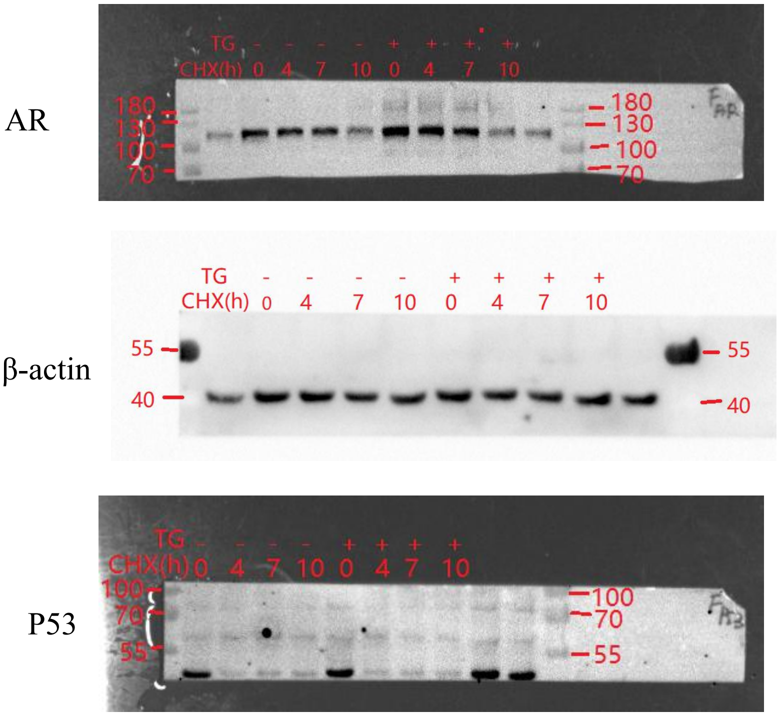

Figure2 e

AR

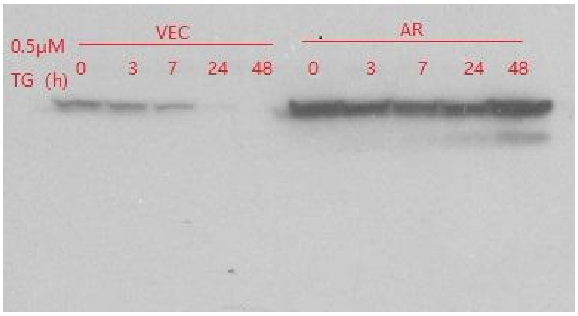

AR
(Longer expo)

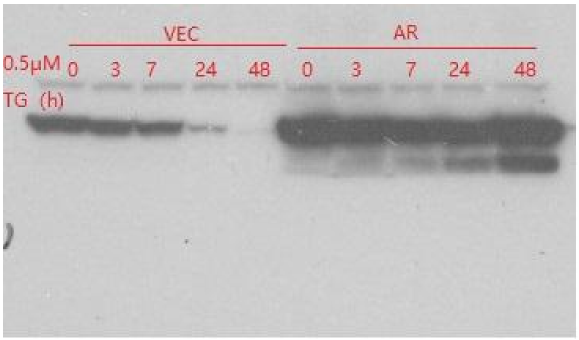

CHOP

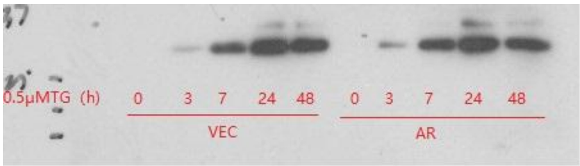

GAPDH

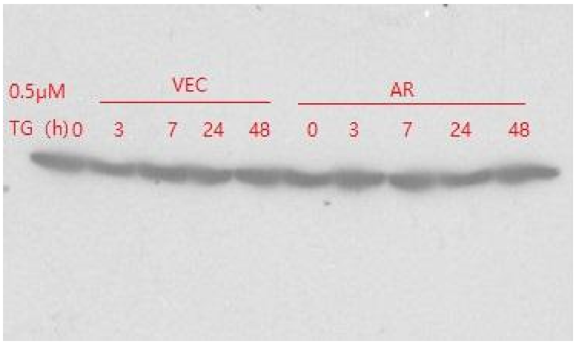

Figure 3 a

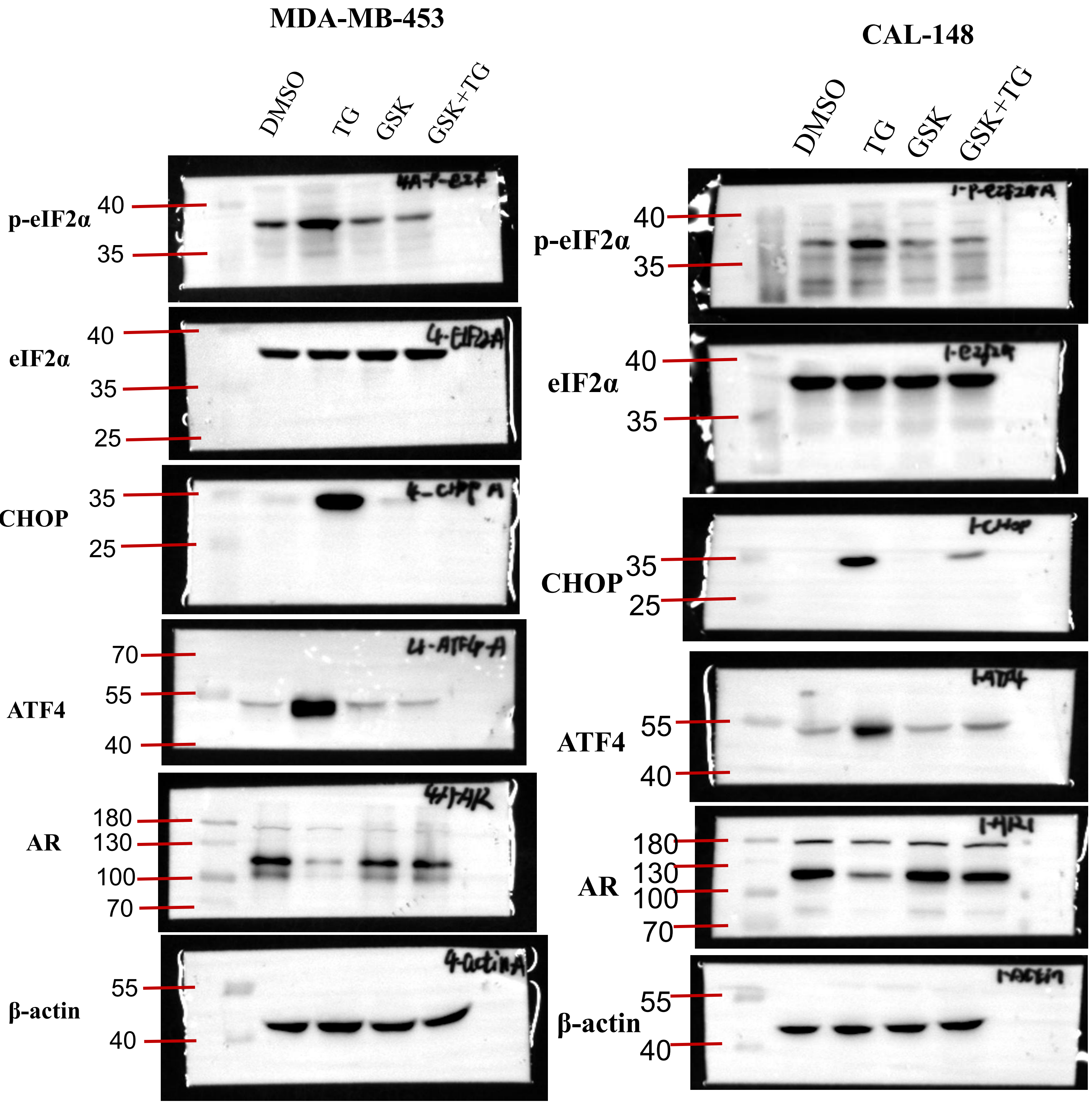
MDA-MB-453
DMSO
TG
GSK
GSK+TG
p-eIF2α
40
35
eIF2α
40
35
25
CHOP
35
25
ATF4
70
55
40
AR
180
130
100
70
β-actin
55
40
CAL-148
DMSO
TG
GSK
GSK+TG
p-eIF2α
40
35
eIF2α
40
35
CHOP
35
25
ATF4
55
40
AR
180
130
100
70
β-actin
55
40

Figure 3 a

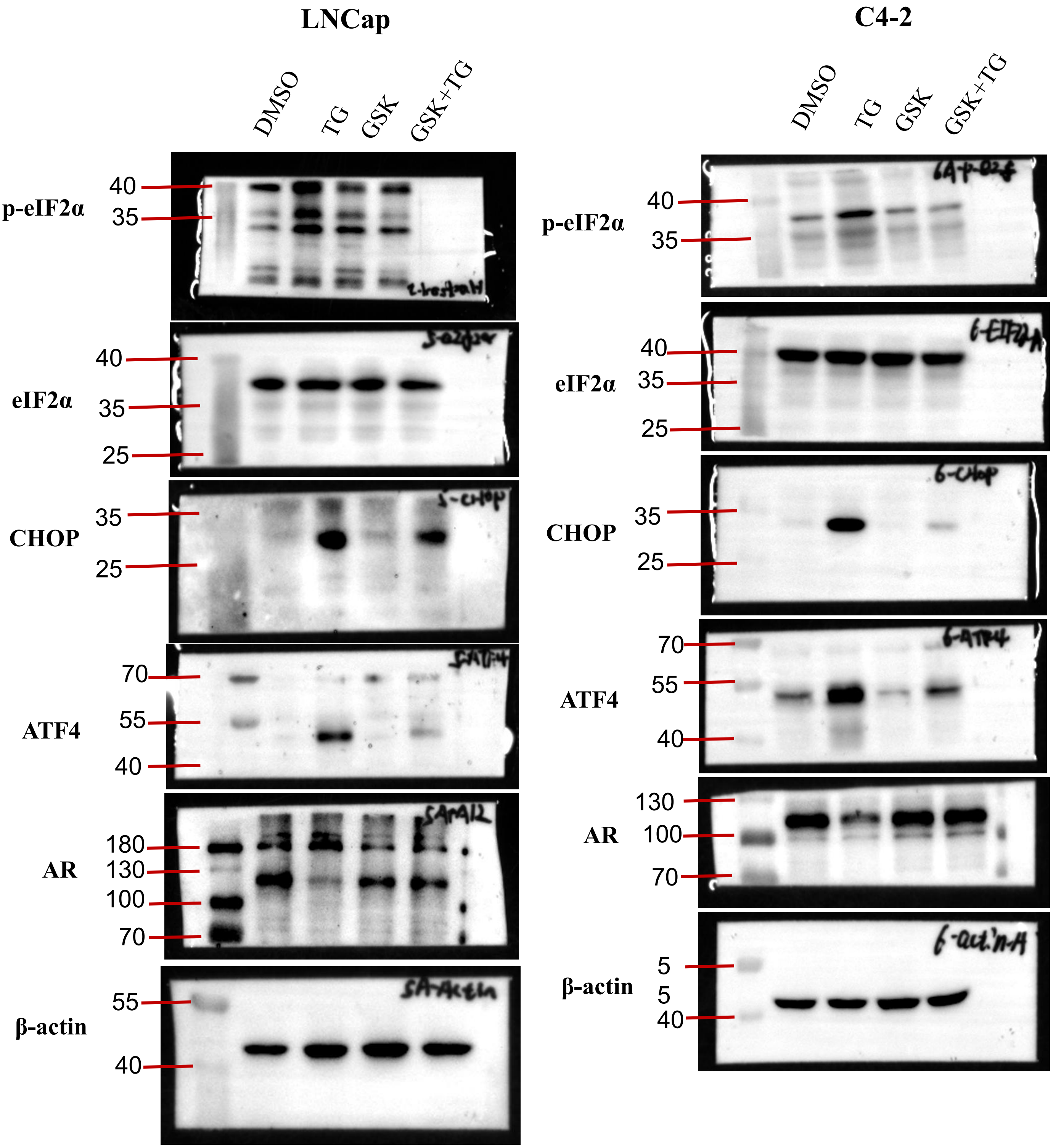

LNCap
C4-2
DMSO
TG
GSK
GSK+TG
p-eIF2α
eIF2α
CHOP
ATF4
AR
β-actin
40
35
25
70
55
180
130
100
5

Figure 3 b

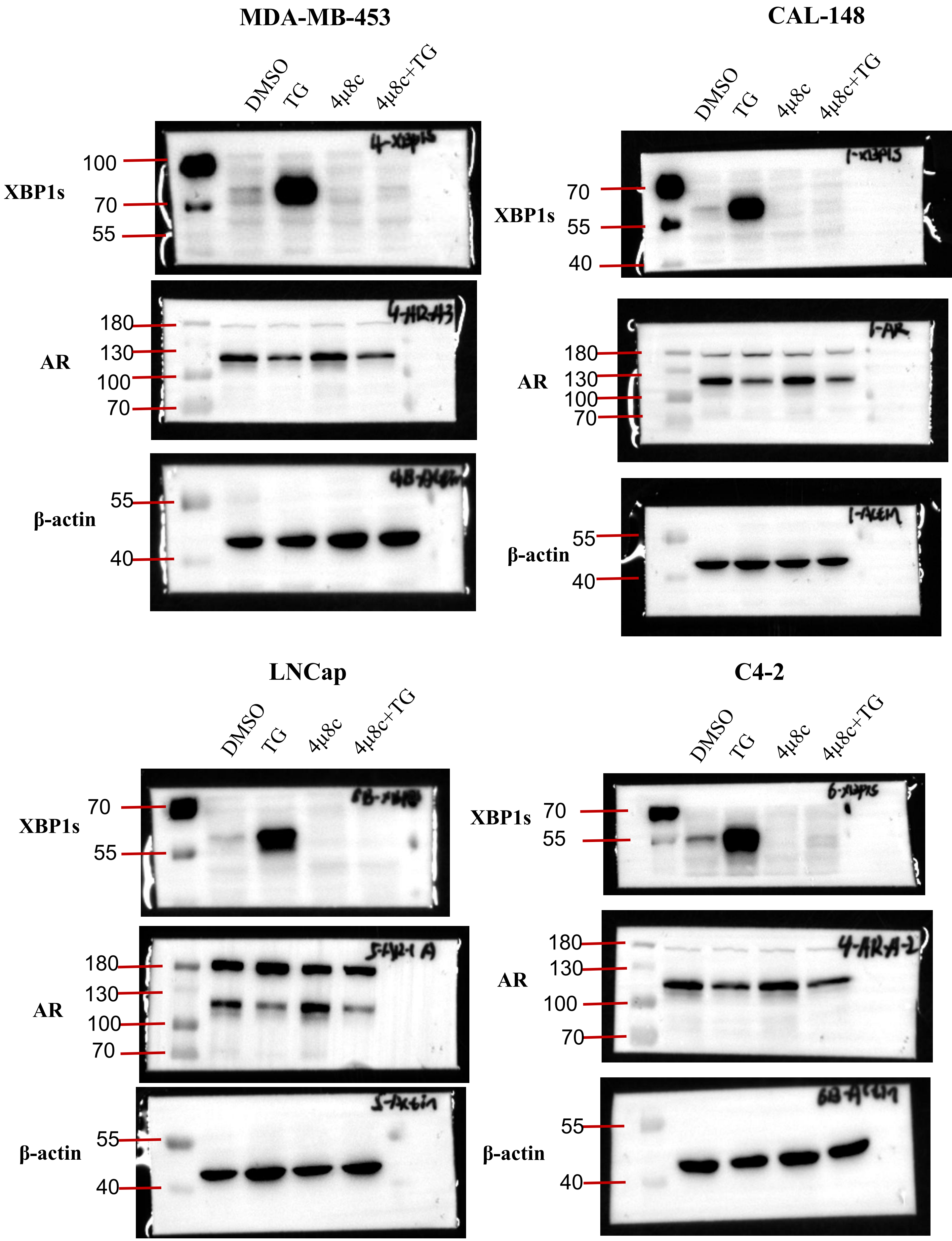
MDA-MB-453
DMSO
TG
4μ8c
4μ8c+TG
XBP1s
100
70
55
AR
180
130
100
70
β-actin
55
40
CAL-148
DMSO
TG
4μ8c
4μ8c+TG
XBP1s
70
55
40
AR
180
130
100
70
β-actin
55
40
LNCap
DMSO
TG
4μ8c
4μ8c+TG
XBP1s
70
55
AR
180
130
100
70
β-actin
55
40
C4-2
DMSO
TG
4μ8c
4μ8c+TG
XBP1s
70
55
AR
180
130
100
70
β-actin
55
40

Figure 3 c

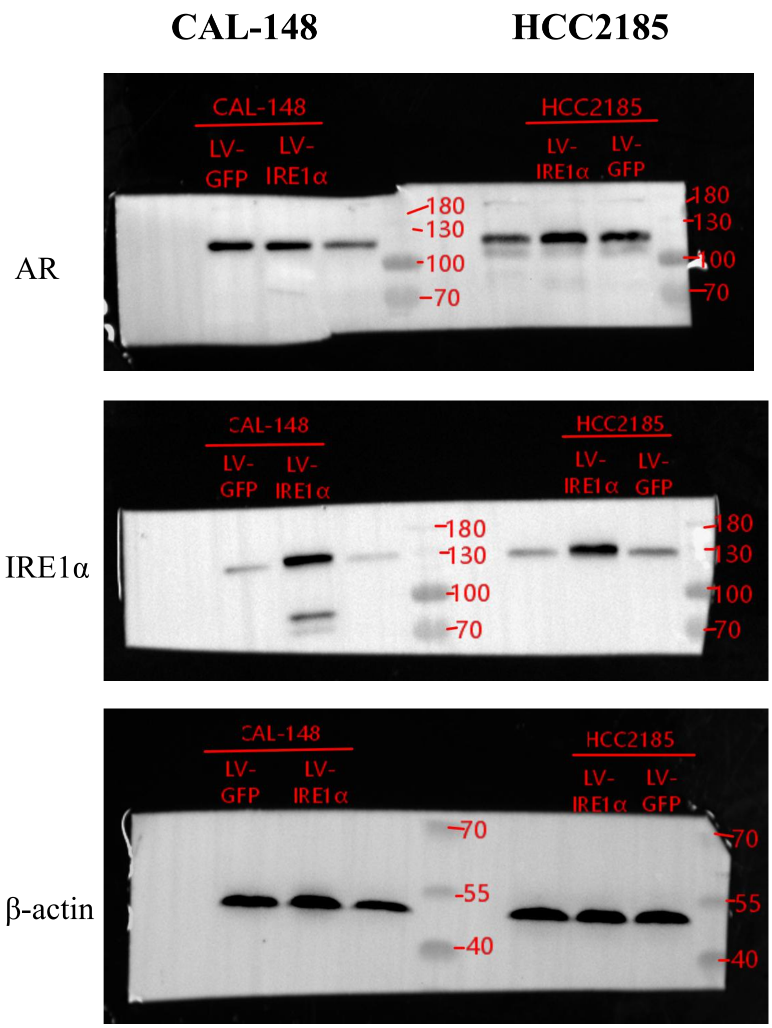
CAL-148
HCC2185
AR
IRE1α
β-actin
CAL-148
LV-
GFP
LV-
IRE1α
HCC2185
LV-
IRE1α
LV-
GFP
180
130
100
70
40
55

Figure 3 c

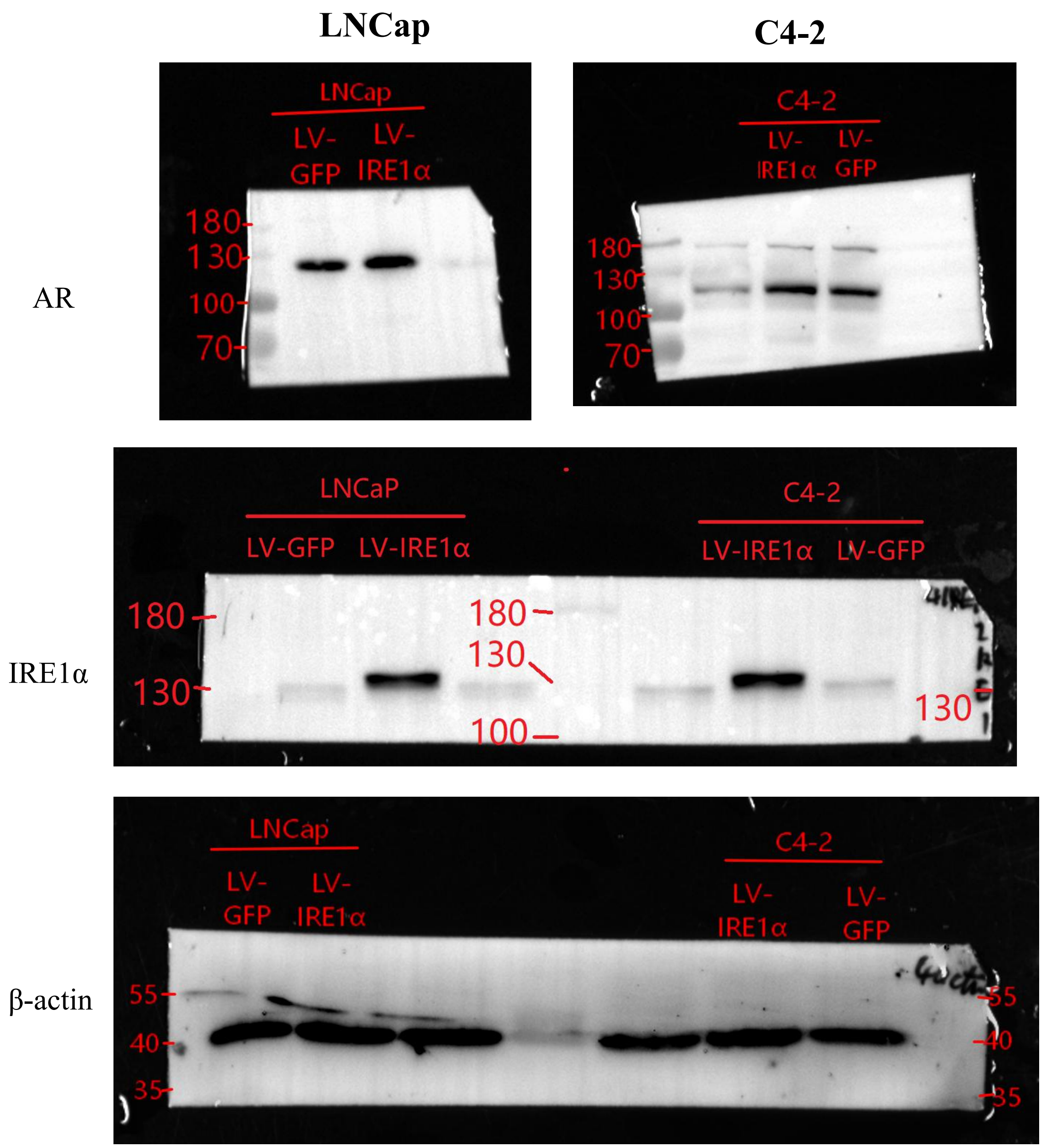

LNCap
C4-2
AR
IRE1α
β-actin
LNCap
LV-GFP
LV-IRE1α
180
130
100
70
C4-2
LV-IRE1α
LV-GFP
180
130
100
70
LNCaP
LV-GFP
LV-IRE1α
C4-2
LV-IRE1α
LV-GFP
180
130
180
130
100
130
LNCap
LV-GFP
LV-IRE1α
C4-2
LV-IRE1α
LV-GFP
55
40
35
55
40
35

Figure 3 d

CAL-148

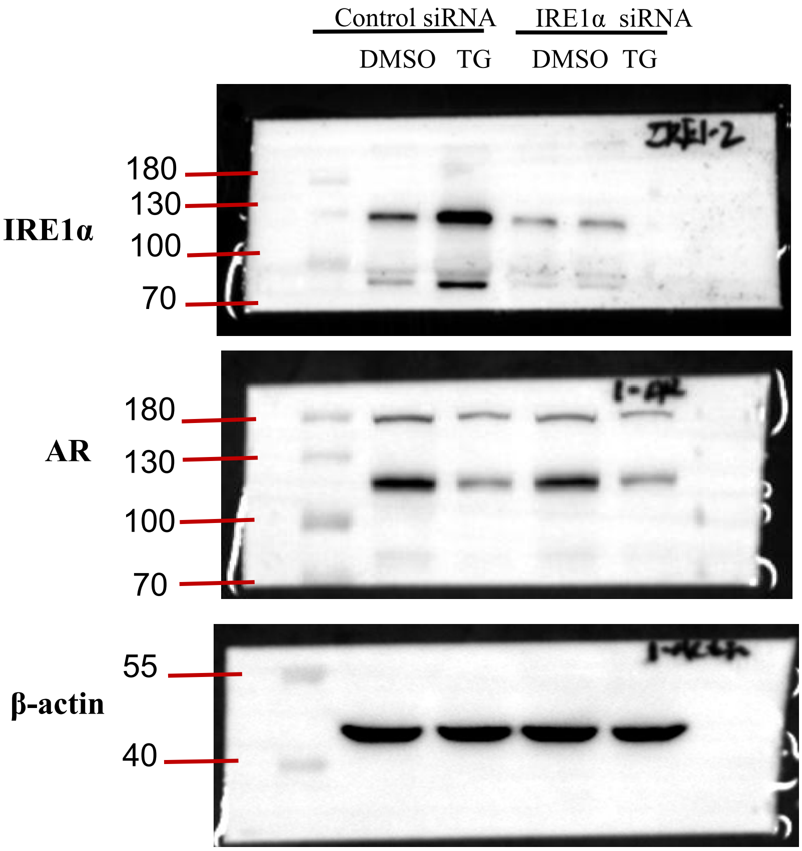

HCC2185

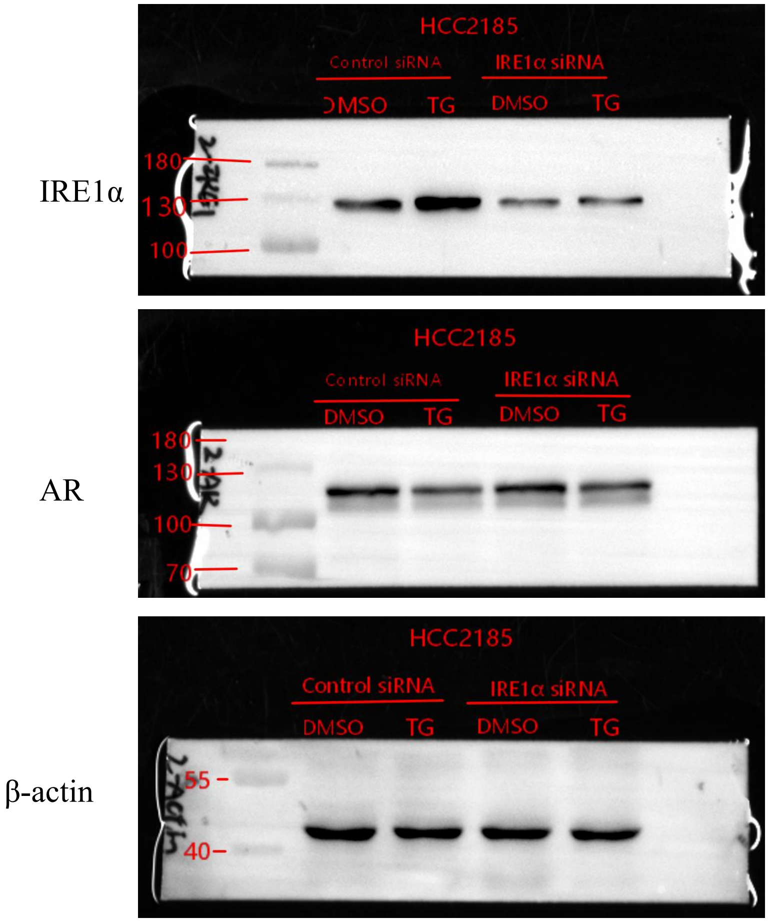

Figure 3 d

C4-2

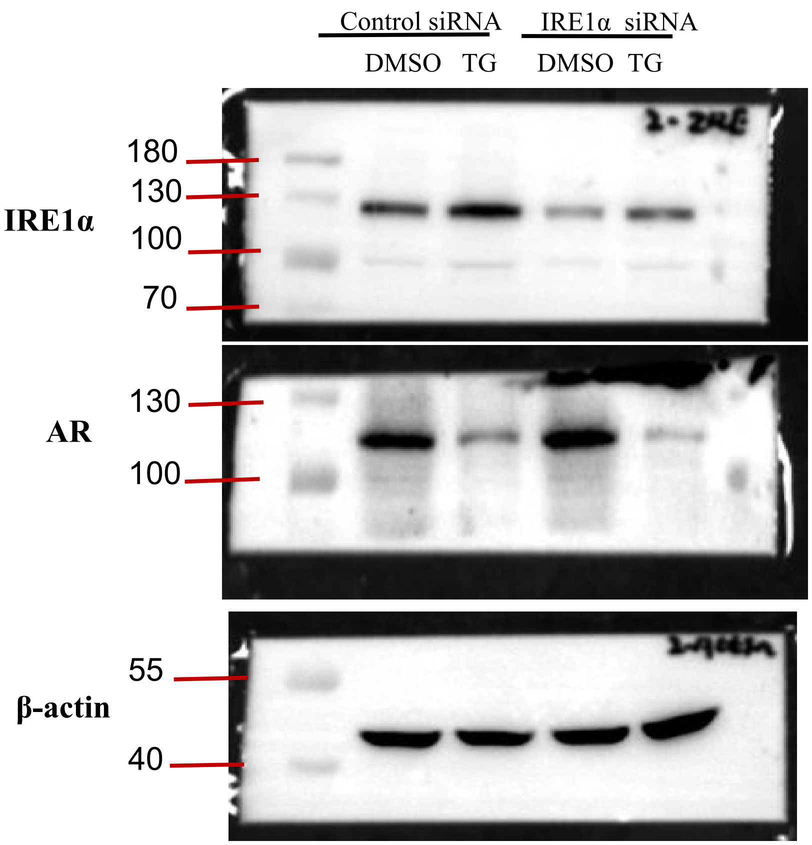

Figure 3 e

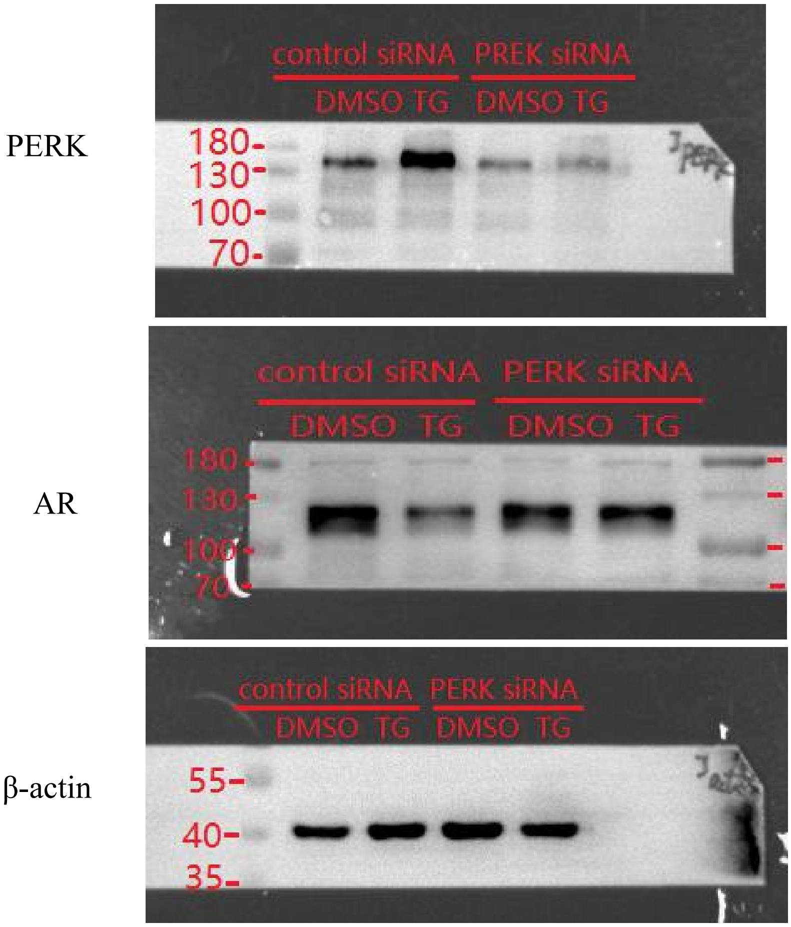

Figure 3 f

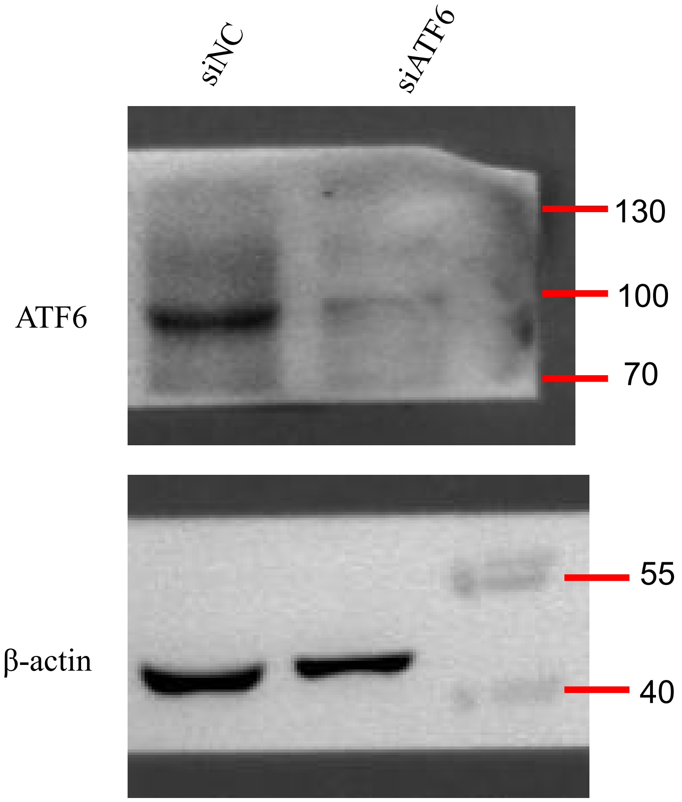

Figure 3 g

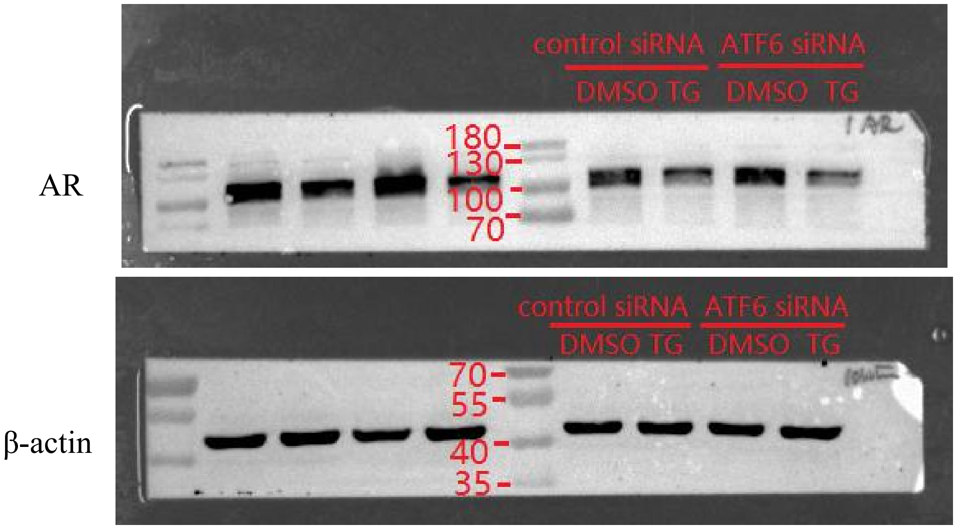

Figure 4 a

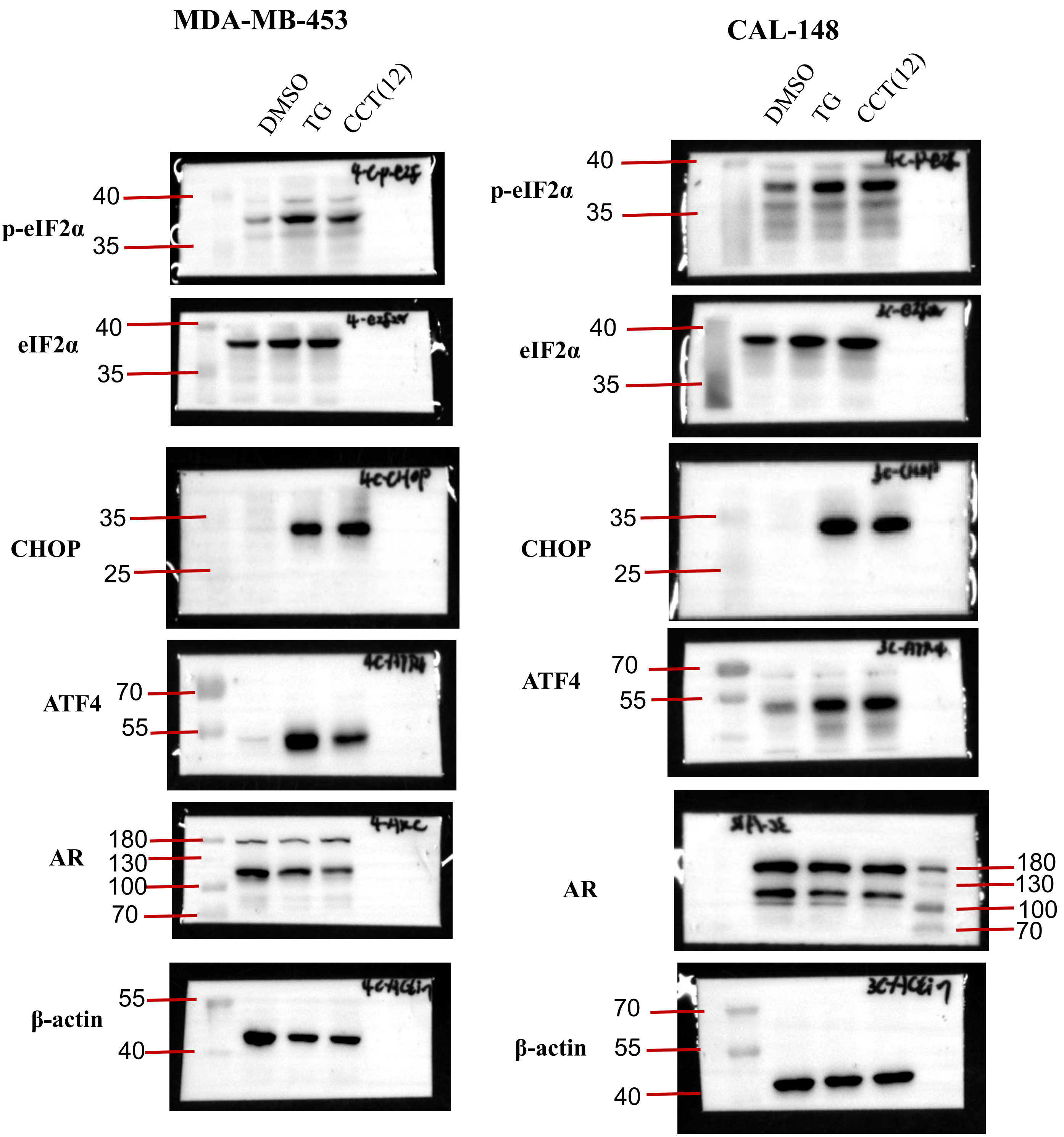

MDA-MB-453
DMSO
TG
CCT(12)
p-eIF2α
40
35
eIF2α
40
35
CHOP
35
25
ATF4
70
55
AR
180
130
100
70
β-actin
55
40
CAL-148
DMSO
TG
CCT(12)
p-eIF2α
40
35
eIF2α
40
35
CHOP
35
25
ATF4
70
55
AR
180
130
100
70
β-actin
70
55
40

Figure 4 b

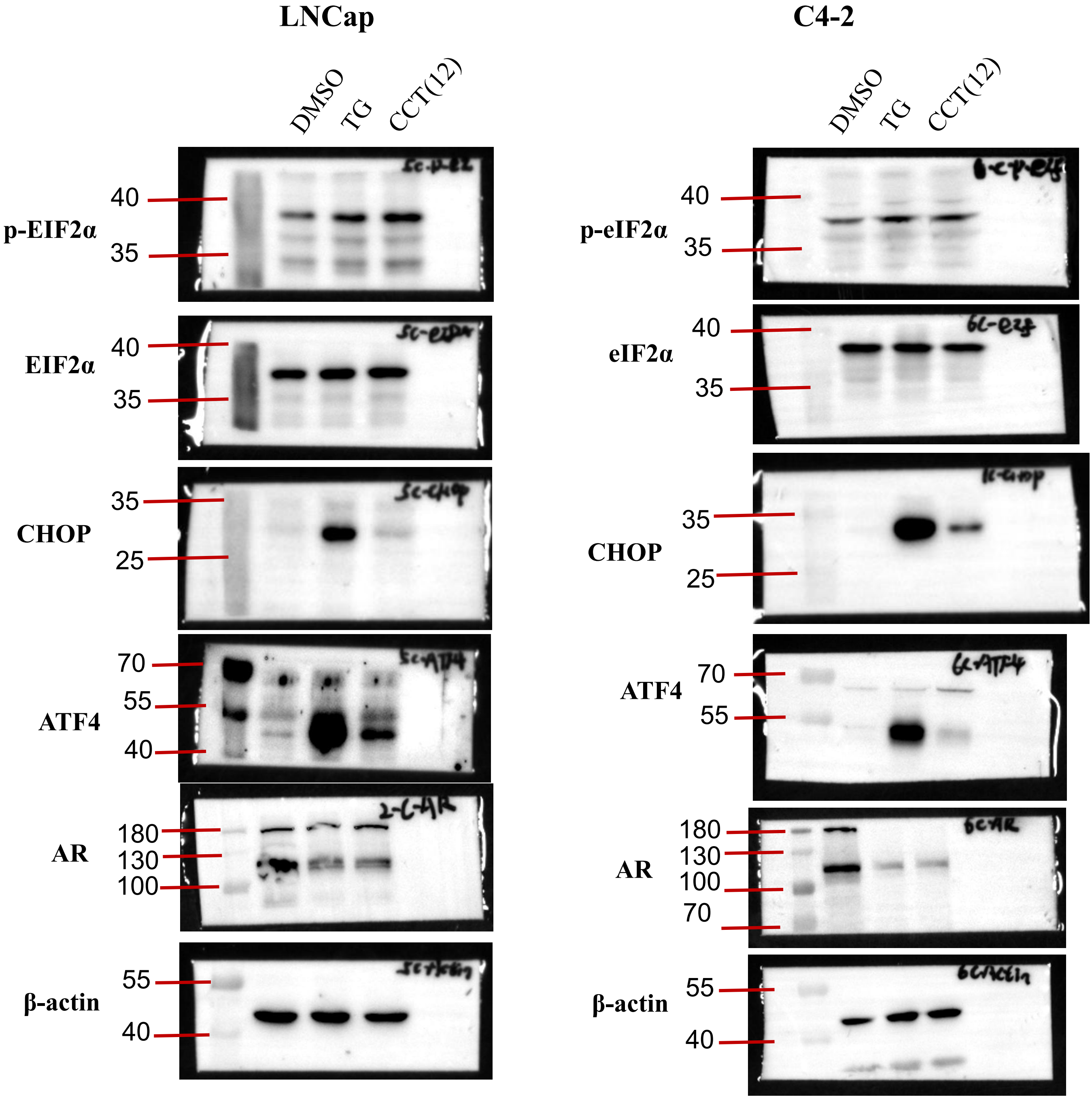
LNCap
C4-2
DMSO
TG
CCT(12)
p-EIF2α
EIF2α
CHOP
ATF4
AR
β-actin
p-eIF2α
eIF2α
40
35
25
70
55
180
130
100

Figure 5 a

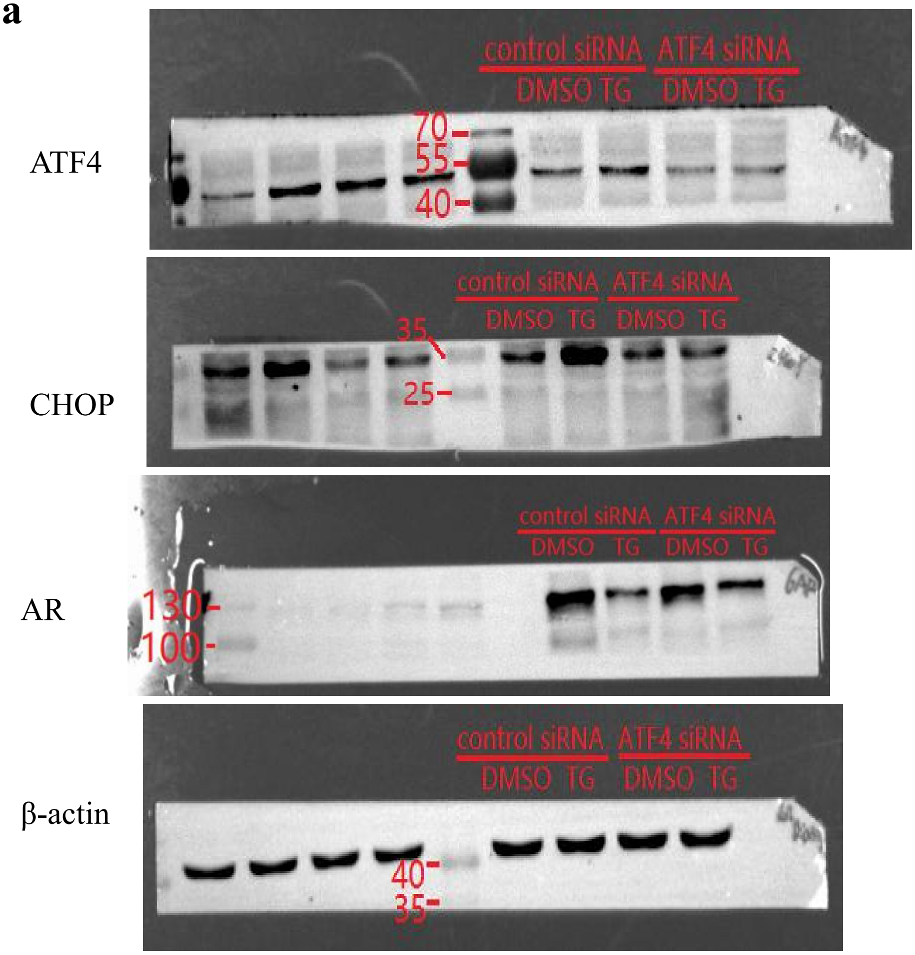

Figure 5 b

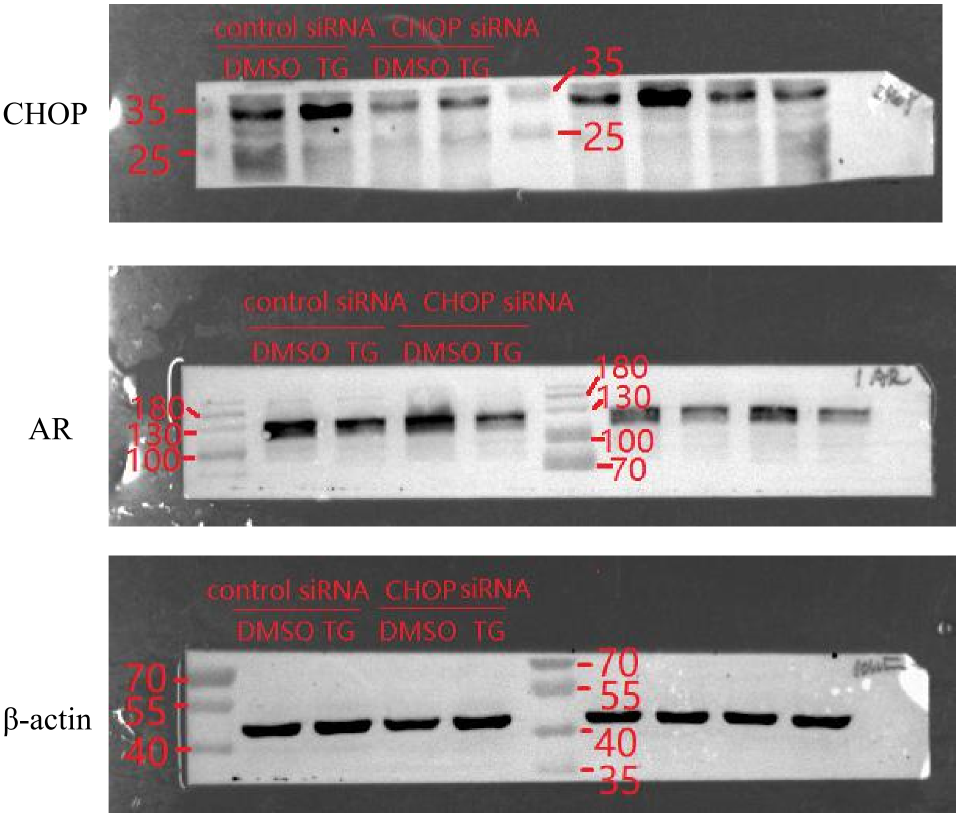

Figure 5 c

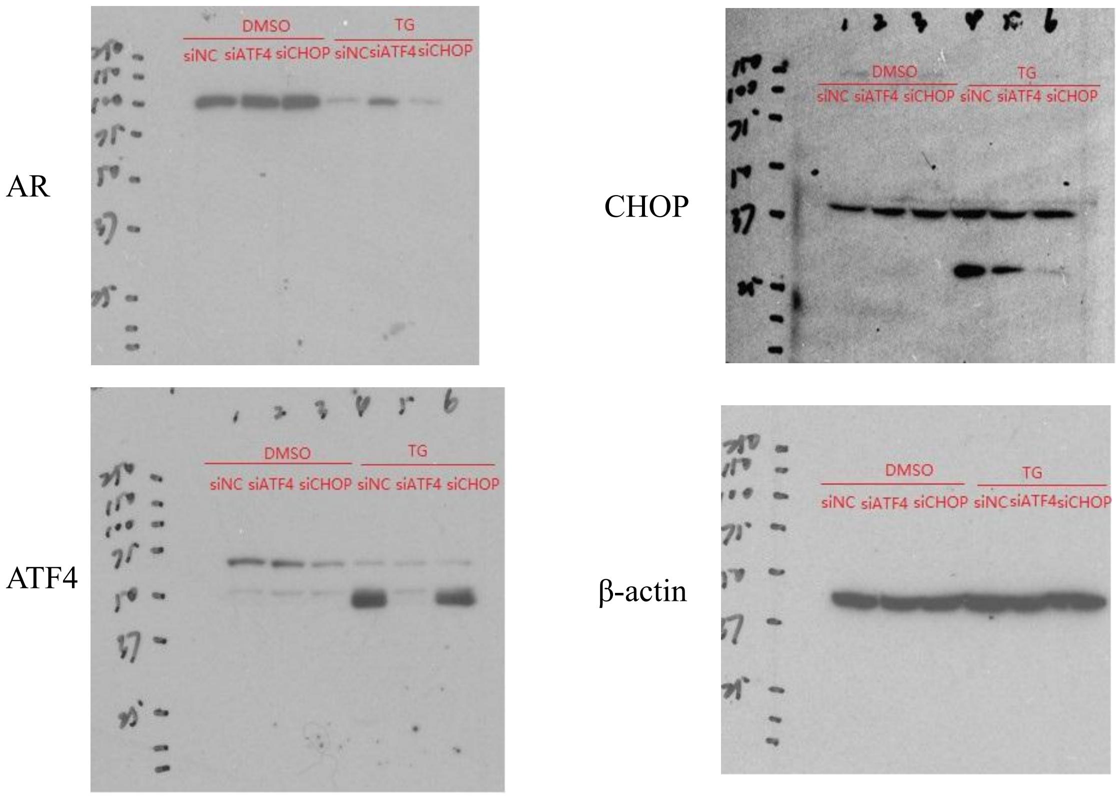

Figure 5 d

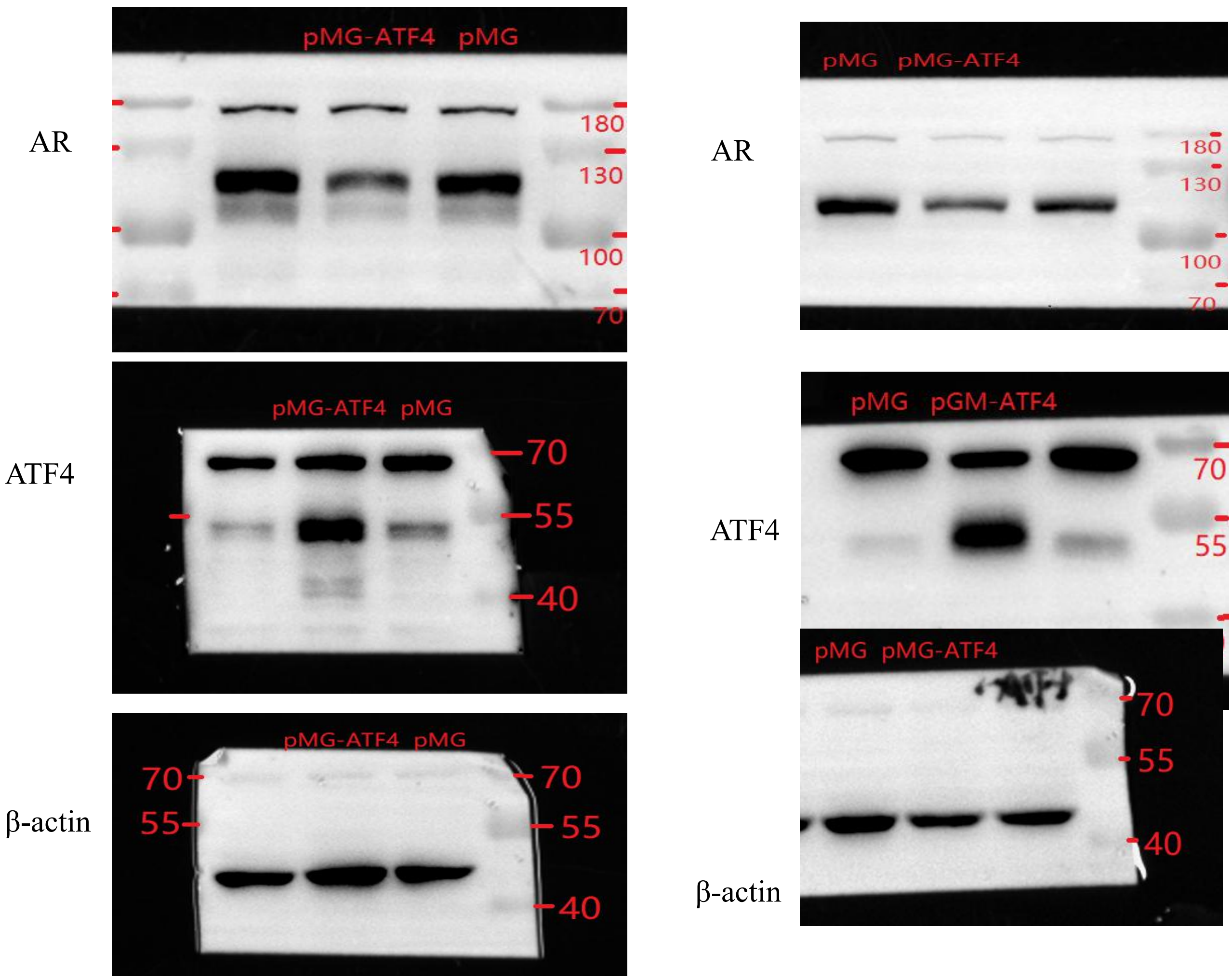

Figure 5 e

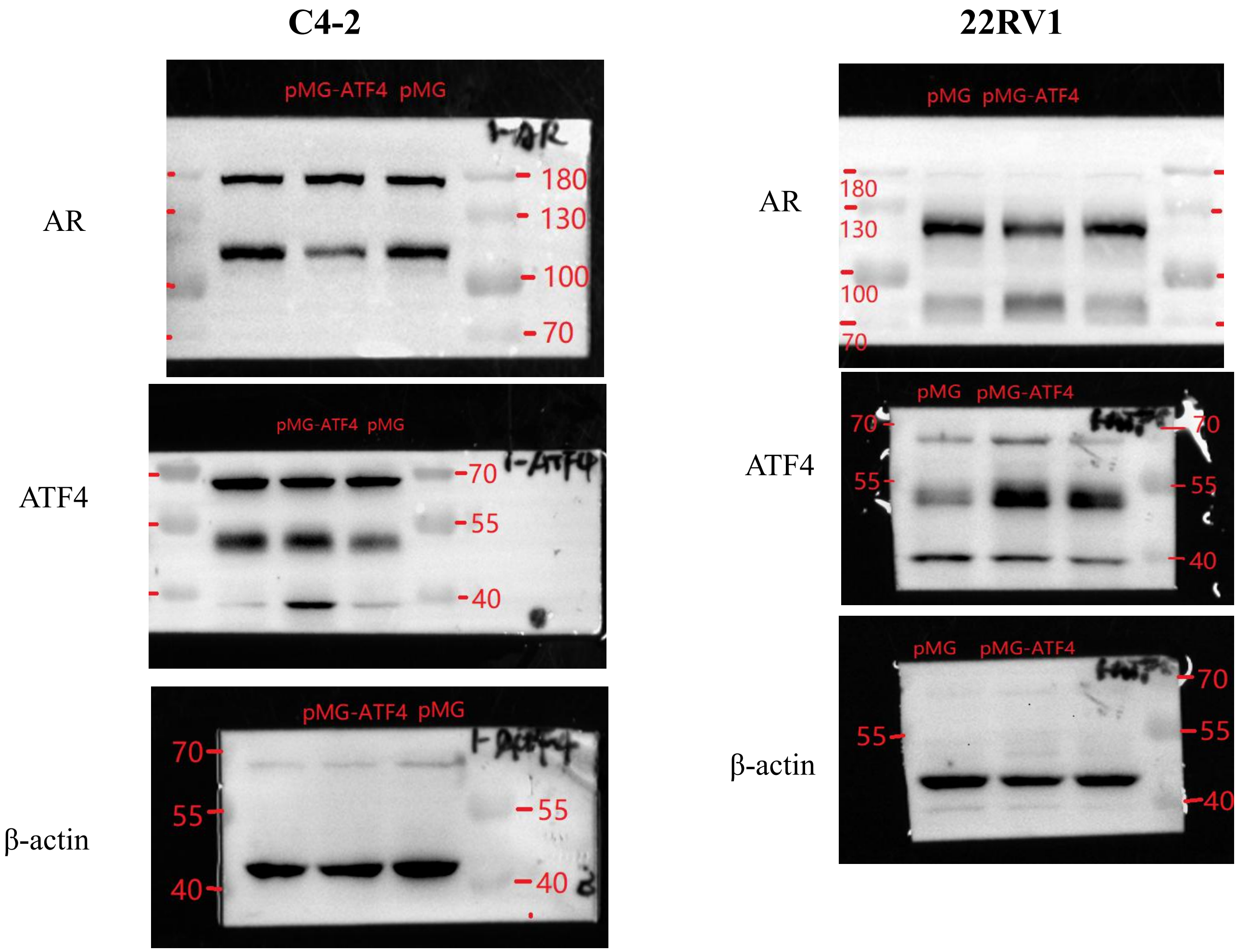
C4-2
22RV1
pMG-ATF4 pMG
AR
180
130
100
70
ATF4
70
55
40
β-actin
pMG pMG-ATF4

Figure 6 b

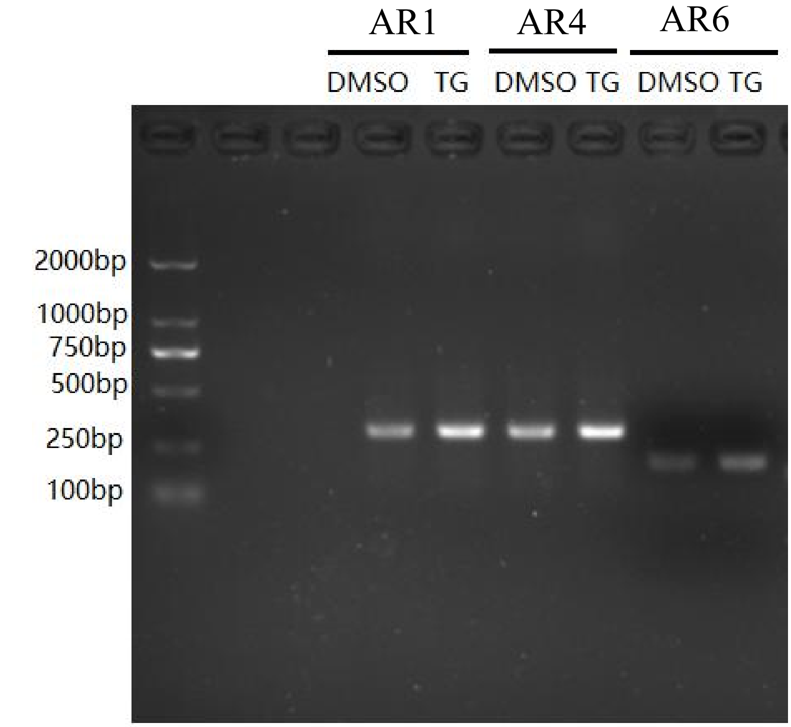

Figure 8 e

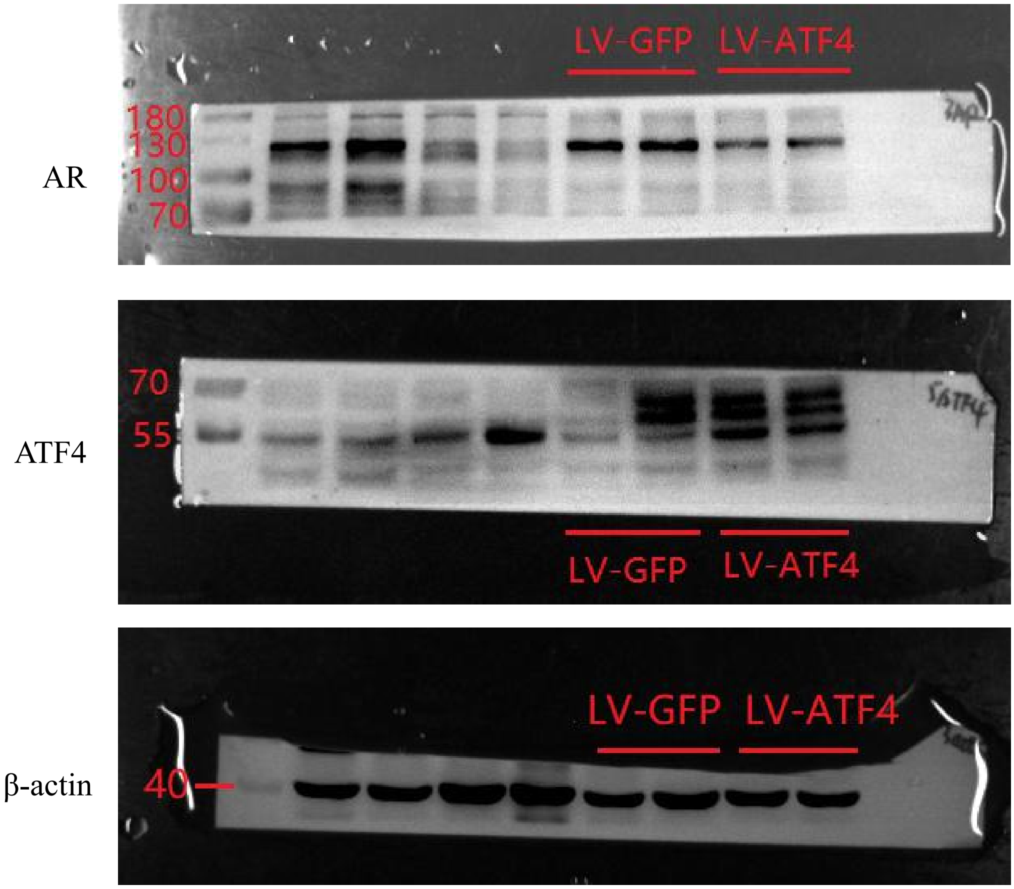

Supplement: Supplementary file 2 — Supplementary [file 41523_2021_370_MOESM2_ESM.pdf]
